# Synthesis of alnustone-like diarylpentanoids via a 4 + 1 strategy and assessment of their potential anticancer activity

**DOI:** 10.55730/1300-0527.3609

**Published:** 2023-10-11

**Authors:** Neslihan ÇELEBİOĞLU, Özlem ÖZDEMİR TOZLU, Hasan TÜRKEZ, Hasan SEÇEN

**Affiliations:** 1 Department of Chemistry, Faculty of Sciences, Atatürk University, Erzurum, Turkiye; 2 Department of Molecular Biology and Genetics, Erzurum Technical University, Erzurum, Turkiye; 3 Department of Medical Biology, Faculty of Medicine, Atatürk University, Erzurum, Turkiye

**Keywords:** 1, 5-Diarylpentanoid, cytotoxic activity, breast cancer, prostate cancer, glioblastoma, anticancer effect

## Abstract

Twelve compounds with a 1,5-diaryl-1-penten-3-one structure were synthesized and their cytotoxic activities were evaluated. The 1,5-diaryl-1-penten-3-one compounds were obtained via in situ enaminations of 4-phenyl-2-butanone and 4-(4-hydroxyphenyl)-2-butanone in the presence of pyrrolidine-AcOH, followed by condensation with six different benzaldehydes. The synthesized compounds were tested for their cytotoxic activity against human glioblastoma (U87-MG), breast (MCF-7), and prostate (PC-3) cancer cell lines. Some of the novel compounds exhibited remarkable cytotoxic action, especially against MCF-7 cancer cells.

## 1. Introduction

**Figure 1:**
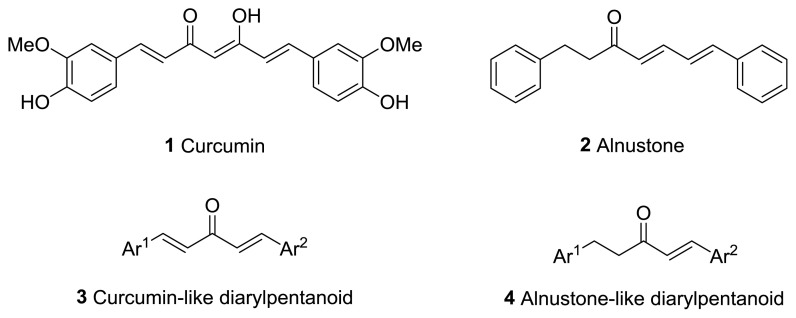
Curcumin (**1**), alnustone (**2**), and related diarylpentanoids (**3**, **4**).

Diarylheptanoids, a special group of natural products, are ArC_7_Ar structured compounds bearing aryl groups at positions C-1 and C-7. These compounds contain C=C, C=O, and OH groups in the heptane chain [1,2]. Natural diarylheptanoids have a great variety of biological and pharmaceutical properties and are therefore recognized as promising therapeutic agents [3,4]. Curcumin (**1**), found in the rhizomes of *Curcuma longa* at a ratio of 3%–5%, is the most widely known natural diarylheptanoid and is the main component of turmeric, a dietary ingredient (Figure 1). Numerous studies have been performed on the biological effects of curcumin (**1**), which include antiinflammatory, immunomodulatory, antibacterial, antiischemic, anticarcinogenic, hepatoprotective, nephroprotective, hypoglycemic, and antirheumatic properties [5].

Diarylpentanoids ArC_5_Ar are two carbonless analogs of diarylheptanoids. Most of these compounds, which have been discovered in recent years, constitute a small group of natural products. To date, nearly 30 diarylpentanoid compounds with different structures have been isolated from nature, their structures have been elucidated, and the biological activities of some of them have been investigated. The isolation and biological activities of 20 diarylpentanoid compounds were described for the first time in a review article by our group in 2017 [6]. Natural diarylpentanoids have been shown to have a wide range of biological effects including anticancer, antiviral, antioxidant, cytotoxic, anti-HIV, nematocidal, antiviral, immunomodulatory, antifeedant, and NO inhibitory effects [6].

Since 1962, when the first natural diarylpentanoid was isolated from nature, they have attracted intense interest as mono-carbonyl analogs of curcumin. In this regard, more than 600 synthetic diarylpentanoid derivatives have been synthesized, and these chemicals have undergone extensive bioactivity studies, with a focus on their anticancer activity. Numerous studies have reported that diarylpentanoid derivatives exhibit a wide range of biological activities including antiviral, antimalarial, and chemopreventive and antitumoral properties [7–11]. The available data support the theory that diarylpentanoid derivatives affect a wide range of biochemical and molecular cascades. In a review article, Moreira et al. [12] examined the structure–activity relationships of synthetic diarylpentanoids in terms of antitumor activity against multiplexed cancer types including glioblastoma (GBM), prostate, and breast cancers. The meta-hydroxyl and adjacent dimethoxyl groups containing diarylpentanoids were found to be cytotoxic and were considered as novel sources of novel anticancer agents [13]. The vast majority of the synthetically prepared diarylpentanoids whose activities have been investigated are 1,4-pentadiene-3-one structured curcumin-like compounds (Figure 1) [12].

Alnustone (**2**), a natural diarylheptanoid, has attracted interest due to its wide range of biological properties, such as its antiinflammatory, antiemetic, antihepatotoxic, and anticancer properties (Figure 1) [14].

In a previous study, it was shown that alnustone-like compounds exhibit stronger antitumor activity than tamoxifen and paclitaxel, which were used as reference drug compounds against the MCF-7 cell line [15].

Artamenone, 1,5-bis-(4-hydroxyphenyl)-2-penten-3-one, a natural diarylheptanoid [16,17], is similar to alnustone (**2**) due to its ArCH_2_CH_2_C(O)CH=CHAr pharmacophore group.

Brain tumor incidence has increased over the past few decades [18]. An estimated 308,102 people received a primary brain or spinal cord tumor diagnosis globally in 2020 [19]. The microglial cell subtype known as astrocytes is the source of the solid tumor known as GBM, which develops in the brain or spinal cord [20]. The capacity of GBM to invade healthy tissues due to the maintenance of continuous angiogenesis makes it the most aggressive type of brain tumor. With a global incidence of fewer than 10 per 100,000 people, GBM is also the most common primary brain tumor, accounting for about 15% of all primary brain tumors in adults [21–23]. GBM also has poor prognosis and a higher fatality rate than other types of brain tumors [24].

In 2020, breast cancer was considered the most frequently diagnosed cancer worldwide. Since 2.3 million new cases of breast cancer (11.7% of the total) were estimated globally in that year, the incidence of breast cancer has overtaken that of lung cancer. Additionally, it contributed to 685,000 deaths from cancer in 2020 and was the sixth highest cause of cancer mortality globally [25,26]. The prevalence of this malignancy has dramatically grown in recent decades, particularly in some areas such as Europe. The determination of risk factors and primary prevention measures to reduce exposure are crucial components of controlling this trend, since the incidence of breast cancer is anticipated to continue rising in the future [27]. Similarly, as one of the most prevalent cancers in men, prostate cancer affects about one in six men, making it a serious public health concern. According to the World Health Organization (WHO), with 1,414,259 new cases worldwide, prostate cancer was the second most common illness and the fifth largest cause of cancer-related deaths among men [28].

**Figure 2:**
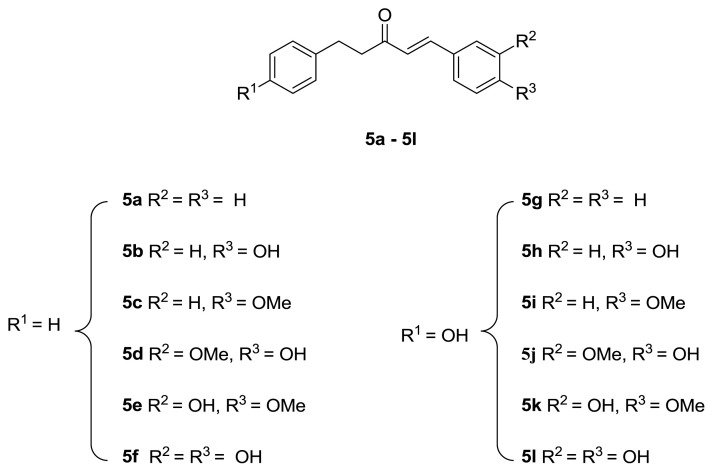
The synthesized alnustone-like diarylpentanoids (**5a**–**5l**).

In the literature, intensive studies have been carried out on the synthesis and bioactivities of curcumin-like diarylpentanoids. However, there are no such studies on alnustone-type diarylpentanoids. In this study, we present a methodology for the synthesis of alnustone-type diarylpentanoids containing ArCH_2_CH_2_C(O)CH=CHAr pharmacophore groups in addition to assessing their cytotoxic activity against the U87-MG, MCF-7, and PC-3 cancer cell lines (Figure 2).

## 2. Results and discussion

**Figure 3:**
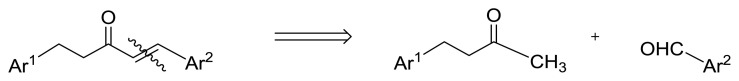
Retrosynthetic analysis of 1,5-diaryl-pent-1-en-3-ones **5a**–**5l**.

Retrosynthetic analysis carried out to synthesize the 1,5-diaryl-1-penten-3-one structures **5a**–**5l** showed that these compounds can be obtained by combining 4-aryl-2-butanones (4 C) with aryl aldehydes (1 C) by condensation under suitable conditions (4 + 1 = 5) (Figure 3).

**Figure 4:**
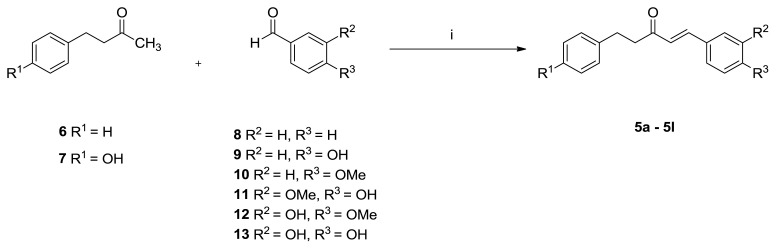
Syntheses of compounds **5a**–**5l**: i) pyrrolidine, AcOH, dry Et_2_O or dry THF, 0 °C, N_2_ atm, 30 min; and then rt, 48–60 h, ~25%–53%.

As shown in Figure 3, the target compounds can be obtained by the condensation of benzaldehydes with 4-aryl-2-butanones. However, this condensation process cannot be carried out by classical Claisen–Schmidt condensation because self-condensation and the Cannizaro reaction can occur under strong basic conditions. In our previous studies [15,29–31], alnustone itself and alnustone-type diarylheptanoids were successfully synthesized by the condensation of 4-aryl-2-butanones and cinnamaldehydes in the presence of AcOH and pyrrolidine (via in situ enamination). In light of this, similar reaction conditions were applied for the synthesis of 1,5-diaryl-1-penten-3-ones **5a**–**5l**. For this purpose, AcOH and pyrrolidine were added to 4-phenylbutan-2-one (**6**) and 4-(4-hydroxyphenyl)butan-2-one (**7**) in dry diethyl ether (Et_2_O) or tetrahydrofuran (THF) at 0 °C and stirred for 30 min. Benzaldehydes were then added and stirred for 48–60 h. Thus, 12 target molecules (**5a**–**5l**) in the structure of the 1,5-diaryl-1-penten-3-ones were obtained in yields ranging from 25% to 53% (Figure 4).

The structures of the synthesized compounds (**5a**–**5l**) were elucidated based on hydrogen-1 NMR (^1^H NMR), carbon-13 NMR (^13^C NMR), high-resolution mass spectrometry (HRMS), and microanalysis. In the ^1^H NMR spectra of the synthesized compounds, all of the H-C(1) and H-C(2) hydrogens resonated as an AX system. The H-C(1) and H-C(2) hydrogens were a conjugated α,β-unsaturated alkene system. Therefore, while the H-C(1) hydrogens representing the A part of the AX system resonated at δ = 7.58–7.46 ppm (downfield) as a doublet, the H-C(2) hydrogens representing the X system resonated between δ = 6.74 and 6.56 ppm (upfield) as a doublet. The *J*_1,2_ values ranged between 15.5 and 16.4 Hz, indicating that the alkene structures in all of the compounds were formed as the *E* configuration (trans), as generally expected. The H_2_C(4) and H_2_C(5) hydrogens arose as an A_2_B_2_ system in the aliphatic region between δ = 3.02 and 2.90 ppm. While the phenyl rings resonated as multiplets in the aromatic region, 4-substituted phenyls resonated as an AA’XX’ system and the 3,4-disubstituted phenyls arose as ABX systems in the aromatic region.

As with the ^1^H NMR spectra, the ^13^C NMR spectra of the 1,5-diaryl-1-penten-3-one compounds were in complete agreement with the structure. Namely, the C-3 ketone carbons resonated at δ = 201.7–199.6 ppm, C(1) carbons resonated at δ = 144.5–141.9 ppm, C(2) carbons resonated at δ = 126.4–122.4 ppm, C(5) carbons resonated at δ = 30.6–29.4 ppm, and C(4) carbons resonated at δ = 42.9–41.9 ppm. The carbons belonging to the Ar^1^ and Ar^2^ ring systems also resonated in the olefinic/aromatic region in accordance with their structures.

GBM (U87-MG), breast cancer (MCF-7), prostate carcinoma (PC-3), and lung epithelium (HPAEpiC) cell lines were used to assess the cytotoxic action potential of compounds **5a**–**5l**. The cells were grown in a single layer in flasks in appropriate medium containing 10% fetal bovine serum (FBS), 1% penicillin/streptomycin, and 1% L-glutamine at 37 °C and 5% CO_2_ to maintain the pH balance. Cells that had reached 70%–80% confluence were used in the experiments.

The 3-(4,5-dimethylthiazol-2-yl)-2,5-diphenyltetrazolium bromide (MTT) method is a widely used colorimetric method for the quantitative determination of cell viability. It is based on the reduction of the tetrazolium ring of the yellow dye MTT by living cells. As a result of the reduction, the tetrazolium ring breaks down to form a dark blue-violet formazan compound. Cell viability is then determined spectrophotometrically. The viability of untreated cells is assumed to be 100% and the viability of treated cells is determined as a percentage (%) according to those cells.

To each 96-well plate, 2 × 10^4^ cells were seeded and treated with 6 different concentrations (3.125, 6.25, 12.5, 25, 50, and 100 mg/L) of the 12 tested compounds, and then the plates were incubated in a CO_2_ oven at 37 °C for 48 h. At the end of the incubation, 10 μL of the MTT mixture was added to each well using a pipette, which was then gently mixed for 1 min on a circular mixer. The cells were incubated in a CO_2_ oven at 37 °C for 3–4 h. The formazan that formed as a result of the incubation was seen as dark crystals at the bottoms of the wells. The culture medium was carefully aspirated from each well without dispersing the cell layer, and then 100 μL of dimethyl sulfoxide (DMSO) was added to each well and gently stirred on an orbital stirrer to dissolve the formazan crystals. Each sample was read at a wavelength of 570 nm in a spectrophotometer and absorbance values were obtained. A wavelength of 630 nm was used as the reference wavelength.

Percent viability and cytotoxicity values for each dose administered were calculated according to the following formulas:

% Viability = (absorbance of the sample/absorbance of the control) × 100
% Cytotoxicity = [1 – (absorbance of the sample/absorbance of the control)] × 100

The IC_50_ dose, as the concentration of inhibitor that caused a 50% decrease in cell viability, was calculated using Excel regression analysis.

The cytotoxic activities of diarylpentanoids **5a**–**5l** against human GBM (U87-MG), breast cancer (MCF-7), prostate cancer (PC-3), and healthy HPAEpiC cells are presented in Table 1.

Compounds **5a**, **5b**, **5d**, **5e**, **5g**, **5h**, and **5i** exerted cytotoxic activity below 30 μM. The IC_50_ values against three different cancer cells indicated that these molecules can be considered as broad-spectrum anticancer agents. Of the 12 compounds examined, 7 compounds (**5a**, **5b**, **5d**, **5e**, **5g**, **5h**, and **5i**) showed cytotoxicity against MCF-7 cells and 3 compounds (**5b**, **5d**, and **5i**) showed cytotoxicity against PC-3 cells below an IC_50_ of < 30 μM. Compared to the doxorubicin (DOX) used as a reference, all of the compounds were less active against the GBM U87-MG cell line compared to the others. However, three of the compounds were more active than DOX against the MCF-7 cell line.

**Figure 5:**
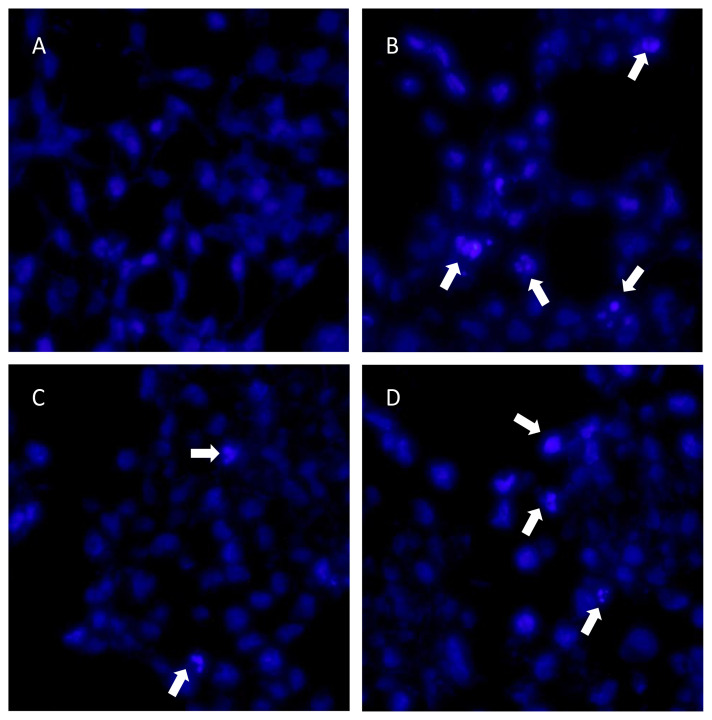
Fluorescent staining of the nuclei in MCF-7 cells by Hoechst 33258. The cells were treated with an IC_50_ concentration of **5a** or **5i** for 48 h. (A) Untreated cells, (B) H_2_O_2_-treated cells, (C) **5a-**treated cells, and (D) **5i-**treated cells. Arrows indicate apoptotic cells with fragmented nuclei. Magnification: 400×.

**Figure 6:**
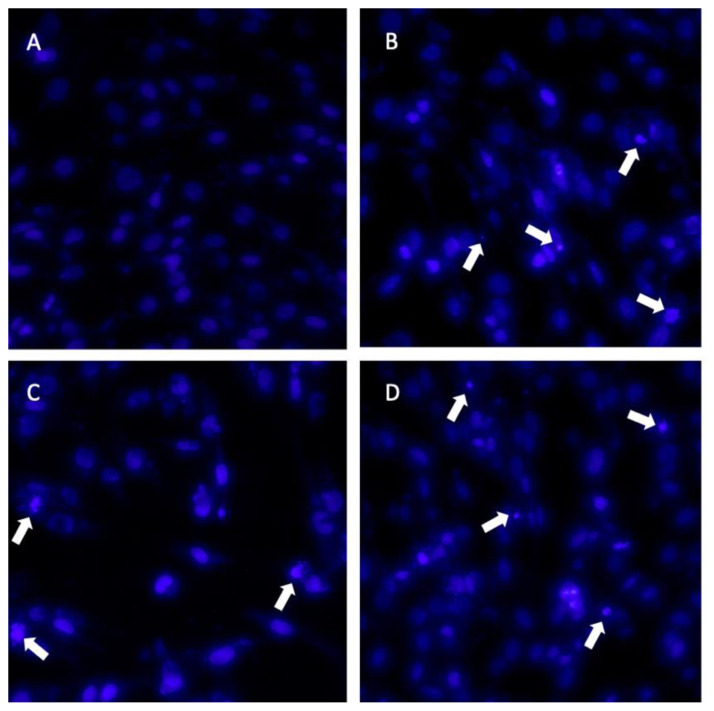
Fluorescent staining of the nuclei in U87-MG cells by Hoechst 33258. The cells were treated with an IC_50_ concentration of **5a** or **5i** for 48 h. (A) Untreated cells, (B) H_2_O_2_**-**treated cells, (C) **5a-**treated cells, and (D) **5i-**treated cells. Arrows indicate apoptotic cells with fragmented nuclei. Magnification: 400×.

Cell morphological characteristics were examined via the fluorescent Hoechst 33258 staining approach. The analysis revealed that there were some alterations in the fluorescent staining characteristics of the treated cells with novel diarylpentanoids **5a**–**5l**. Hoechst 33258 staining revealed considerable morphological alterations in the nuclear chromatin. The nuclei in the untreated group were stained a less brilliant blue and the color was uniform. After 48 h of treatment at the IC_50_ concentrations of compounds **5a** and **5i**, the blue emission light in the apoptotic cells was considerably brighter than in the control cells. Many compound-treated cells also had condensed chromatin, and some had the shape of apoptotic bodies, which is one of the basic features of apoptotic cells (Figures 5 and 6). In line with these findings, it was previously reported that the diarylpentanoid BP-M345 exerted potential as an anticancer drug, showing its antiproliferative activity by preventing mitosis through microtubule disruption and inducing cancer cell death [32]. Likewise, diarylpentanoid derivatives have led to intrinsic apoptosis in cancer cells by upregulating proapoptotic proteins such as Bad and Bax and downregulating prosurvival proteins such as Bcl-2 and Bcl-xL [33]. Their possible molecular targets were associated with the mitogen-activated protein kinase/extracellular signal-regulated kinase (MAPK/ERK), signal transducers and activators of transcription (STAT), and nuclear factor kappa-light-chain-enhancer of activated B cells (NF-κB) signaling pathways that are involved in cancer development and progression [34].

Selectivity, which is the most relevant parameter in determining in vitro anticancer potential, was used to determine the efficacy of the compounds [35,36]. The selectivity index (SI) for each compound was expressed as SI = IC_50_ for normal cells (HPAEpiC)/IC_50_ for the cancer cell lines (U87-MG, PC-3, and MCF-7) (Table 2).

The HPAEpiC cell line was used as a noncancerous cell line for evaluating the cytotoxicity of the newly synthesized compounds, since epithelial tissues are widespread throughout the body and these cells turnover rapidly and mutations naturally accumulate throughout life. Moreover, epithelial tissue is also considered as the most common site for the development of cancers. In terms of the SI, three of the compounds had good selectivity against the MCF-7 cell line (**5a**: 7.45, **5j**: 2.67, and **5i**: 2.52; reference DOX: 4.38). It is especially noteworthy that compound **5a** had a better SI value against MCF-7 cells than the reference DOX. For PC-3 cells, two compounds (**5j**: 2.90 and **5l**: 2.14) showed high selectivity.

## 3. Conclusion

In this study, 12 diarylpentanoids in the structures of 1,5-diaryl-1-penten-3-one were realized by condensation of two 4-aryl-2-butanones and six benzaldehydes. The obtained compounds were tested against the U87-MG, MCF-7, and PC-3 cancer cell lines. The synthesized compounds showed broad-spectrum antitumor activity, especially against the MCF-7 cell line. Moreover, compound **5a** showed a better SI value against MCF-7 cells than reference drug DOX. Alnustone and 1,5-diphenyl-1-penten-3-one both contain nonsubstituted phenyl rings and they are structurally very similar to each other. This similarity indicates that the 1,5-diphenyl-1-penten-3-one structure is a very potent pharmacophore group. Collectively, these findings suggest that the alnustone-like diarylpentanoids might be considered as novel sources of effective broad-spectrum anticancer agents and deserve further research.

## 4. Experimental

All of the reactions were carried out under a nitrogen atmosphere and monitored by thin-layer chromatography (TLC) and NMR spectroscopy. The ^1^H NMR and ^13^C NMR spectra were recorded with 400 (100) MHz Bruker (Bruker Corp., Billerica, MA, USA) and Varian (Varian Medical Systems, Palo Alto, CA, USA) instruments. Exchangeable hydrogens or carbons were marked with the same letters. Elemental analyses were recorded using a LECO CHNS-932 elemental analyzer (LECO Corporation, St. Joseph, MI, USA) and HRMS spectra were recorded using an Agilent 6530 LC-MS QTOF (Agilent Technologies, Santa Clara, CA, USA). Melting points were measured using a Gallenkamp melting point device.

### 4.1. General synthetic procedures for the 1,5-diaryl-1-penten-3-ones ((*E*)-1,5-diphenylpent-1-en-3-one (5a))

4-Phenylbutan-2-one (**6**) (2.04 g, 13.74 mmol) was dissolved in 5 mL of dry Et_2_O at 0 °C under a nitrogen atmosphere. A solution of pyrrolidine (1.07 g, 1.25 mL, 15.11 mmol) and AcOH (0.90 g, 0.86 mL, 15.11 mmol) in 5 mL of dry Et_2_O was added dropwise to the reaction medium under the same reaction conditions for 10 min. After stirring for 30 min, the temperature was reduced to 0 °C and a solution of benzaldehyde (**8**) (1.45 g, 13.74 mmol) in 5 mL of Et_2_O was added dropwise over 30 min. The reaction mixture was stirred at room temperature for 48 h by monitoring with TLC. To the reaction medium, 10 mL of 1 M HCl solution was added. The crude product was extracted with 2 × 50 mL of Et_2_O and the organic phases were combined. The organic phases were washed with 2 × 30 mL of H_2_O, dried over Na_2_SO_4_, and the solvent was evaporated. The crude product was purified by column chromatography on a silica gel column using 6:94 EtOAc-hexane and then crystallized with EtOAc-hexane to give (*E*)-1,5-diphenylpent-1-en-3-one (**5a**) (1.81 g, 56%). R_f_ (AcOEt/hexanes 1:9): 0.6. White solid. Mp 56–57 °C. ^1^H NMR (400 MHz, CDCl_3_): d 7.55 (d, 1H, H-1, *J*_1_,_2_ = 16.2 Hz), 7.55–7.52 (m, 2H, H-2′/6′), 7.41–7.39 (m, 3H, H-3′/4′/5′), 7.33–7.22 (m, 5H, H-2″/3″/4″/5″/6″), 6.74 (d, 1H, H-2, *J*_1_,_2_ = 16.2 Hz), 3.02 (bs, A_2_B_2_, 4H, CH_2_CH_2_) ppm; ^13^C NMR (100 MHz, CDCl_3_): d 199.6 (C-3), 143.0 (C-1), 141.5 (C-1″), 134.7 (C-1′), 130.7 (C-4′^a^), 129.2 (C-2′/6′^b^), 128.8 (C-3′/5′^b^), 128.6 (C-2″/6″^b^), 128.5 (C-3″/5″^b^), 126.4 (C-4″/2^a^), 42.7 (C-4), 30.4 (C-5) ppm; Anal. Calcd. for C_17_H_16_O (MW 236.31): C, 86.40; H, 6.82%. Found: C, 86.01; H, 6.90%. HR-ESI-MS: [M + H]^+^ 237.1295; calc. 237.1279.

The ^1^H NMR and ^13^C NMR data of compound **5a** were in good agreement with the data given in the literature [37].

### 4.2. (*E*)-1-(4-hydroxyphenyl)-5-phenylpent-1-en-3-one (5b)

The synthetic procedure described above for **5a** was applied using 4-phenyl-2-butanone (**6**) and 4-hydroxybenzaldehyde (**9**) as a reagent and Et_2_O as a solvent for 60 h to give **5b** in a yield of 31%. Chromatography was performed using EtOAc/hexanes at a ratio of 20:80. R_f_ (AcOEt/hexanes 3:7): 0.8. Light yellow solid. Mp 136–137 °C. Anal. Calcd. for C_17_H_16_O_2_ (MW 252.31): C, 80.93; H, 6.39%. Found: C, 80.79; H, 6.51%. HR-ESI-MS: [M + H]^+^ 253.1250; calc. 253.1228.

### 4.3. (*E*)-1-(4-methoxyphenyl)-5-phenylpent-1-en-3-one (5c)

The synthetic procedure described above for **5a** was applied using 4-phenyl-2-butanone (**6**) and 4-methoxybenzaldehyde (**10**) as a reagent and Et_2_O as a solvent for 48 h to give **5c** in a yield of 72%. Chromatography was performed using EtOAc/hexanes in a ratio of 4:96. R_f_ (AcOEt/hexanes 1:9): 0.6. White solid. Mp 95–96 °C; Anal. Calcd. for C_18_H_18_O_2_ (MW 266.34): C, 81.17; H, 6.81%. Found: C, 81.10; H, 6.87%. HR-ESI-MS: [M + H]^+^ 267.1398; calc. 267.1385.

The ^1^H NMR and ^13^C NMR data of compound **5c** were in good agreement with the data given in the literature [38].

### 4.4. (*E*)-1-(4-hydroxy-3-methoxyphenyl)-5-phenylpent-1-en-3-one (5d)

The synthetic procedure described above for **5a** was applied using 4-phenyl-2-butanone (**6**) and 4-hydroxy-3-methoxybenzaldehyde (**11**) as a reagent and Et_2_O as a solvent for 48 h to give **5d** in a yield of 61%. Chromatography was performed using EtOAc/hexanes in a ratio of 20:80. R_f_ (AcOEt/hexanes 3:7): 0.7. Yellow solid. Mp 89–90 °C. Anal. Calcd. for C_18_H_18_O_3_ (MW 282.34): C, 76.57; H, 6.43%. Found: C, 76.11; H, 6.57%. HR-ESI-MS: [M + H]^+^ 283.1353; calc. 283.1334.

The ^1^H NMR and ^13^C NMR data of compound **5d** were in good agreement with the data given in the literature [39].

### 4.5. (*E*)-1-(3-hydroxy-4-methoxyphenyl)-5-phenylpent-1-en-3-one (5e)

The synthetic procedure described above for **5a** was applied using 4-phenyl-2-butanone (**6**) and 3-hydroxy-4-methoxybenzaldehyde (**12**) as a reagent and Et_2_O as a solvent for 48 h to give **5e** in a yield of 44%. Chromatography was performed using EtOAc/hexanes in a ratio of 20:80. R_f_ (AcOEt/hexanes 3:7): 0.6. Brown solid. Mp 94–95 °C. Anal. Calcd. for C_18_H_18_O_3_ (MW 282.34): C, 76.57; H, 6.43%. Found: C, 76.77; H, 6.42%. HR-ESI-MS: [M + H]^+^ 283.1354; calc. 283.1334.

### 4.6. (*E*)-1-(3,4-dihydroxyphenyl)-5-phenylpent-1-en-3-one (5f) [40]

The synthetic procedure described above for **5a** was applied using 4-phenyl-2-butanone (**6**) and 3,4-dihydroxybenzaldehyde (**13**) as a reagent and THF as a solvent for 48 h to give **5f** in a yield of 53%. Chromatography was performed using EtOAc/hexanes in a ratio of 30:70. R_f_ (AcOEt/hexanes 3:7): 0.6. Brown solid. Mp 167–168 °C. Anal. Calcd. for C_17_H_16_O_3_ (MW 268.31): C, 76.10; H, 6.01%. Found: C, 75.99; H, 6.17%. HR-ESI-MS: [M + H]^+^ 269.1193; calc. 269.1177.

### 4.7. (*E*)-5-(4-hydroxyphenyl)-1-phenylpent-1-en-3-one (5g)

The synthetic procedure described above for **5a** was applied using 4-hydroxyphenyl-2-butanone (**7**) and benzaldehyde (**8**) as a reagent and Et_2_O as a solvent for 48 h to give **5g** in a yield of 37%. Chromatography was performed using EtOAc/hexanes in a ratio of 15:85. R_f_ (AcOEt/hexanes 2:8): 0.6. Light yellow solid. Mp 96–97 °C. Anal. Calcd. for C_17_H_16_O_2_ (MW 252.31): C, 80.93; H, 6.39%. Found: C, 81.23; H, 6.54%. HR-ESI-MS: [M + H]^+^ 253.1243; calc. 253.1228.

### 4.8. (*E*) 1,5-bis(4-hydroxyphenyl)pent-1-en-3-one (5h)

The synthetic procedure described above for **5a** was applied using 4-hydroxyphenyl-2-butanone (**7**) and 4-hydroxybenzaldehyde (**9**) as a reagent and THF as a solvent for 48 h to give **5h** in a yield of 20%. Chromatography was performed using EtOAc/hexanes in a ratio of 25:75. R_f_ (AcOEt/hexanes 3:7): 0.46. Yellow solid. Mp 146–147 °C. Anal. Calcd. for C_17_H_16_O_3_ (MW 268.31): C, 76.10; H, 6.01%. Found: C, 76.00; H, 6.19%. HR-ESI-MS: [M + H]^+^ 269.1175; calc. 269.1177.

### 4.9. (*E*)-5-(4-hydroxyphenyl)-1-(4-methoxyphenyl)pent-1-en-3-one (5i)

The synthetic procedure described above for **5a** was applied using 4-hydroxyphenyl-2-butanone (**7**) and 4-methoxybenzaldehyde (**10**) as a reagent and THF as a solvent for 48 h to give **5i** in a yield of 36%. Chromatography was performed using EtOAc/hexanes in a ratio of 20:80. R_f_ (AcOEt/hexanes 3:7): 0.6. Light yellow solid. Mp 83–84 °C. Anal. Calcd. for C_18_H_18_O_3_.0.4 H_2_O (MW 289.54): C, 74.6; H, 6.59%. Found: C, 74.11; H, 6.42%. HR-ESI-MS: [M + H]^+^ 283.1350^;^ calc. 283.1334.

### 4.10. (*E*)-1-(4-hydroxy-3-methoxyphenyl)-5-(4-hydroxyphenyl)pent-1-en-3-one (5j)

The synthetic procedure described above for **5a** was applied using 4-hydroxyphenyl-2-butanone (**7**) and 4-hydroxy-3-methoxybenzaldehyde (**11**) as a reagent and THF as a solvent for 48 h to give **5j** in a yield of 21%. Chromatography was performed using EtOAc/hexanes in a ratio of 30:70. R_f_ (AcOEt/hexanes 4:6): 0.75. Yellow solid. Mp 131–132 °C. Anal. Calcd. for C_18_H_18_O_4_ (MW 298.34): C, 72.47; H, 6.08%. Found: C, 72.06; H, 6.08%. HR-ESI-MS: [M + H]^+^ 299.1303; calc. 299.1283.

### 4.11. (*E*)-1-(3-hydroxy-4-methoxyphenyl)-5-(4-hydroxyphenyl)pent-1-en-3-one (5k)

The synthetic procedure described above for **5a** was applied using 4-hydroxyphenyl-2-butanone (**7**) and 3-hydroxy-4-methoxybenzaldehyde (**12**) as a reagent and THF as a solvent for 48 h to give **5k** in a yield of 11%. Chromatography was performed using EtOAc/hexanes in a ratio of 1:1. R_f_ (AcOEt/hexanes 4:6): 0.7. Light yellow solid. Mp 130–131 °C. Anal. Calcd. for C_18_H_18_O_4_ (MW 298.34): C, 72.47; H, 6.08%. Found: C, 72.42; H, 5.89%. HR-ESI-MS: [M + H]^+^ 299.1264; calc. 299.1283.

### 4.12. (*E*)-1-(3,4-dihydroxyphenyl)-5-(4-hydroxyphenyl)pent-1-en-3-one (5l)

The synthetic procedure described above for **5a** was applied using 4-hydroxyphenyl-2-butanone (**7**) and 3,4-dihydroxybenzaldehyde (**13**) as a reagent and THF as a solvent for 48 h to give **5l** in a yield of 23%. Chromatography was performed using EtOAc/hexanes in a ratio of 40:60. R_f_ (AcOEt/hexanes 5:5): 0.5. Yellow solid. Mp 202–203 °C. Anal. Calcd. for C_17_H_16_O_4_ (MW 284.31): C, 71.82; H, 5.67%. Found: C, 71.52; H, 5.99%. HR-ESI-MS: [M + H]^+^ 285.1140; calc. 285.1126.

The ^1^H NMR and ^13^C NMR spectra of compounds **5a**–**5l** are given in the supplementary material file as graphical and numerical data.

### 4.13. Cytotoxicity testing

#### 4.13.1. Cell lines and culture conditions

Human GBM (U87-MG), breast cancer (MCF-7), prostate cancer (PC-3), and pulmonary alveolar epithelial cells (HPAEpiC) cell lines from the American Type Culture Collection (ATCC) were used for the cytotoxicity testing of compounds **5a**–**5l**. The cells were grown as a single layer in appropriate medium containing 10% FBS, 1% penicillin/streptomycin, and 1% L-glutamine at 37 °C and 5% CO_2_ atmosphere.

#### 4.13.2. MTT assay

The cytotoxicity analyses of all of the compounds were investigated by determining cell viability based on the colorimetric MTT assay, which was conducted according to the manufacturer’s guide. First, 2 × 10^5^ cells/well were seeded in a 96-well plate and then incubated at 37 °C in a 5% CO_2_ environment for 24 h. The medium was aspirated the following day, and 100 mL of each concentration of the 12 tested compounds (3.125, 6.25, 12.5, 25, 50, and 100 mg/L) were separately added to each well. The plates were then incubated for 48 h at 37 °C with 5% CO_2_. Next, 10 μL of MTT solution was added to each well and the plate was incubated for 4 h. The culture medium was aspirated from the wells without dispersing the cell layer and then 100 μL of DMSO was added to each well and stirred on an orbital stirrer to dissolve the formazan crystals. Each sample was read at a wavelength of 570 nm in a spectrophotometer and absorbance values were obtained. A wavelength of 630 nm was used as the reference. Cell viability was expressed as a percentage compared to the untreated cells. All experiments were performed in triplicate [41,42].

#### 4.13.3. Hoechst 33258 staining

Hoechst 33258 staining was used to assess significant morphological changes in the nuclear chromatin of the cells. Briefly, the cells were seeded on coverslips in a 6-well plate and treated with the IC_50_ concentration of compounds **5a** and **5i**. After 48 h, the cover glasses were washed carefully with PBS, fixed in 4% paraformaldehyde in 0.01 M PBS for 1 h, and washed twice with PBS. The cells were then stained with 20 μg/mL Hoechst 33258 for 10 min. Thereafter, the cells were observed using a Leica DMIRB fluorescence microscope (Leica Microsystems, Wetzlar, Germany). Additionally, 100 μM H_2_O_2_ was used as a positive control.

#### 4.13.4. Statistical analysis

IBM SPSS Statistics 20.0 for Windows (IBM Corp., Armonk, NY, USA) was used for statistical analyses and two-way ANOVA was used to statistically compare data from multiple groups. P < 0.05 was considered statistically significant.

## Supplementary Material

Figure 1 ^1^H-NMR spectrum of (*E*)-1,5-diphenylpent-1-en-3-one (**5a**) (CDCl_3_).

Figure 2 ^13^C-NMR spectrum of (*E*)-1,5-diphenylpent-1-en-3-one (**5a**) (CDCl_3_).

Figure 3 HRMS spectrum of (*E*)-1,5-diphenylpent-1-en-3-one (**5a**). (C_17_H_16_O+H)^+^, Calc: 237.1279.

Figure 4 ^1^H-NMR spectrum of (*E*)-1-(4-hydroxyphenyl)-5-phenylpent-1-en-3-one (**5b**) (CDCl_3_).

Figure 5 **^13^**C-NMR spectrum of (*E*)-1-(4-hydroxyphenyl)-5-phenylpent-1-en-3-one (**5b**) (CDCl_3_).

Figure 6 HRMS spectrum of (*E*)-1-(4-hydroxyphenyl)-5-phenylpent-1-en-3-one (**5b**). (C_17_H_16_O_2_+H)^+^, Calc: 253.1228.

Figure 7 **^1^**H-NMR spectrum of (*E*)-1-(4-methoxyphenyl)-5-phenylpent-1-en-3-one (**5c**) (CDCl_3_).

Figure 8 ^13^C-NMR spectrum of (*E*)-1-(4-methoxyphenyl)-5-phenylpent-1-en-3-one (**5c**) (CDCl_3_).

Figure 9 HRMS spectrum of (*E*)-1-(4-methoxyphenyl)-5-phenylpent-1-en-3-one (**5c**). (C_18_H_18_O_2_+H)^+^, Calc: 267.1385.

Figure 10 ^1^H-NMR spectrum of (*E*)-1-(4-hydroxy-3-methoxyphenyl)-5-phenylpent-1-en-3-one (**5d**) (CDCl_3_).

Figure 11 **^13^**C-NMR spectrum of (*E*)-1-(4-hydroxy-3-methoxyphenyl)-5-phenylpent-1-en-3-one (**5d**) (CDCl_3_).

Figure 12 HRMS spectrum of (*E*)-1-(4-hydroxy-3-methoxyphenyl)-5-phenylpent-1-en-3-one (**5d**). (C_18_H_18_O_3_+H)^+^, Calc: 283.1334.

Figure 13 ^1^H-NMR spectrum of (*E*)-1-(3-hydroxy-4-methoxyphenyl)-5-phenylpent-1-en-3-one (**5e**) (CDCl_3_).

Figure 14 ^13^C-NMR spectrum of (*E*)-1-(3-hydroxy-4-methoxyphenyl)-5-phenylpent-1-en-3-one (**5e**) (CDCl_3_).

Figure 15 HRMS spectrum of (*E*)-1-(3-hydroxy-4-methoxyphenyl)-5-phenylpent-1-en-3-one (**5e**). (C_18_H_18_O_3_+H)^+^, Calc: 283.1334.

Figure 16 ^1^H-NMR spectrum of (*E*)-1-(3,4-dihydroxyphenyl)-5-phenylpent-1-en-3-one (**5f**) (Acetone-d_6_).

Figure 17 ^13^C-NMR spectrum of (*E*)-1-(3,4-dihydroxyphenyl)-5-phenylpent-1-en-3-one (**5f**) (Acetone-d_6_).

Figure 18 HRMS spectrum of (*E*)-1-(3,4-dihydroxyphenyl)-5-phenylpent-1-en-3-one (**5f**). (C_17_H_16_O_3_+H)^+^, Calc: 269.1177.

Figure 19 ^1^H-NMR spectrum of (*E*)-5-(4-hydroxyphenyl)-1-phenylpent-1-en-3-one (**5g**) (CDCl_3_).

Figure 20 ^13^C-NMR spectrum of (*E*)-5-(4-hydroxyphenyl)-1-phenylpent-1-en-3-one (**5g**) (CDCl_3_).

Figure 21 HRMS spectrum of (*E*)-5-(4-hydroxyphenyl)-1-phenylpent-1-en-3-one (**5g**). (C_17_H_16_O_2_+H)^+^, Calc: 253.1228.

Figure 22 ^1^H-NMR spectrum of (*E*)-1,5-bis(4-hydroxyphenyl)pent-1-en-3-one (**5h**) (Acetone-d_6_).

Figure 23 ^13^C-NMR spectrum of (*E*)-1,5-bis(4-hydroxyphenyl)pent-1-en-3-one (**5h**) (Acetone-d_6_).

Figure 24 HRMS spectrum of (*E*)-1,5-bis(4-hydroxyphenyl)pent-1-en-3-one (**5h**). (C_17_H_16_O_3_+H)^+^, Calc: 269.1177.

Figure 25 ^1^H-NMR spectrum of (*E*)-5-(4-hydroxyphenyl)-1-(4-methoxyphenyl)pent-1-en-3-one (**5i**) (Acetone-d_6_).

Figure 26 ^13^C-NMR spectrum of (*E*)-5-(4-hydroxyphenyl)-1-(4-methoxyphenyl)pent-1-en-3-one (**5i**) (Acetoned_6_).

Figure 27 HRMS spectrum of (*E*)-5-(4-hydroxyphenyl)-1-(4-methoxyphenyl)pent-1-en-3-one (**5i**). (C_18_H_18_O_3_+H)^+^, Calc: 283.1334.

Figure 28 ^1^H-NMR spectrum of (*E*)-1-(4-hydroxy-3-methoxyphenyl)-5-(4-hydroxyphenyl)pent-1-en-3-one (**5j**) (Acetone-d_6_).

Figure 29 ^13^C-NMR spectrum of (*E*)-1-(4-hydroxy-3-methoxyphenyl)-5-(4-hydroxyphenyl)pent-1-en-3-one (**5j**) (Acetone-d_6_).

Figure 30 HRMS spectrum of (*E*)-1-(4-hydroxy-3-methoxyphenyl)-5-(4-hydroxyphenyl)pent-1-en-3-one (**5j**). (C_18_H_18_O_4_+H)^+^, Calc: 299.1283.

Figure 31 ^1^H-NMR spectrum of (*E*)-1-(3-hydroxy-4-methoxyphenyl)-5-(4-hydroxyphenyl)pent-1-en-3-one (**5k**) (Acetone-d_6_).

Figure 32 ^13^C-NMR spectrum of (*E*)-1-(3-hydroxy-4-methoxyphenyl)-5-(4-hydroxyphenyl)pent-1-en-3-one (**5k**) (Acetone-d_6_).

Figure 33 HRMS spectrum of (*E*)-1-(3-hydroxy-4-methoxyphenyl)-5-(4-hydroxyphenyl)pent-1-en-3-one (**5k**). (C_18_H_18_O_4_+H)^+^, Calc: 299.1283.

Figure 34 ^1^H-NMR spectrum of (*E*)-1-(3,4-dihydroxyphenyl)-5-(4-hydroxyphenyl)pent-1-en-3-one (**5l**) (Methanol-d_4_).

Figure 35 ^13^C-NMR spectrum of (*E*)-1-(3,4-dihydroxyphenyl)-5-(4-hydroxyphenyl)pent-1-en-3-one (**5l**) (Methanol-d_4_).

Figure 36 HRMS spectrum of (*E*)-1-(3,4-dihydroxyphenyl)-5-(4-hydroxyphenyl)pent-1-en-3-one (**5l**). (C_17_H_16_O_4_+H)^+^, Calc: 285.1126.

**Table 1:** 1H-NMR data of compounds **5a–5l** (δ (ppm); J (Hz)).

Compound	Ar^2^ δ(ppm)	CH_2_CH_2_δ (ppm)	H-C(1) δ (ppm)	H-C(2) δ (ppm)	Ar^1^ δ (ppm)
**5a**	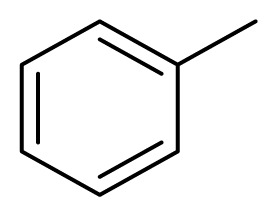 7.33-7.22 (m, 5H)	3.02 (A2B2, bs, 4H)	7.55 (d, 1H, *J* = 16.2)	6.74 (d, 1H, *J* = 16.2)	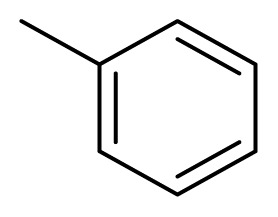 7.55-7.52 (m, 2H) 7.41-7.39 (m, 3H)
**5b**	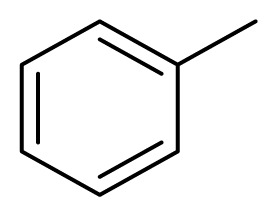 7.32-7.18 (m, 5H)	3.01 (A2B2, bs, 4H)	7.51 (d, 1H, *J* = 16.1)	6.62 (d, 1H, *J* = 16.1)	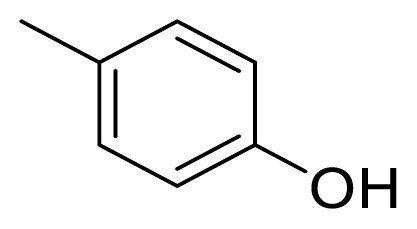 7.43 (d, 2H, *J* = 8.8) 6.87 (d, 2H, *J* = 8.8)
**5c**	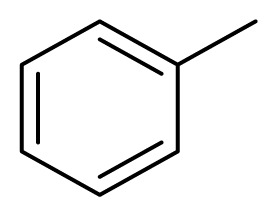 7.32-7.18 (m, 5H)	3.00 (A2B2, bs, 4H)	7.51 (d, 1H, *J* = 16.1)	6.62 (d, 1H, *J* = 16.1)	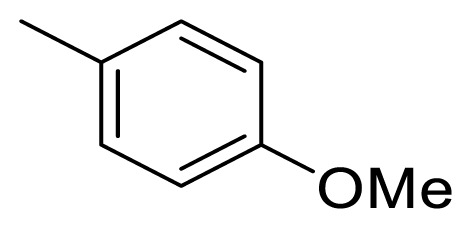 7.48 (d, 2H, *J* = 8.4) 6.90 (d, 2H, *J* = 8.4) 3.84 (s, OMe)
**5d**	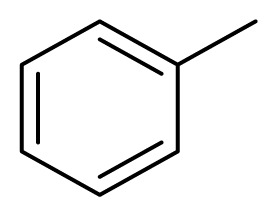 7.32-7.18 (m, 5H)	3.00 (A2B2, bs, 4H)	7.48 (d, 1H, *J* = 16.3)	6.59 (d, 1H, *J* = 16.3)	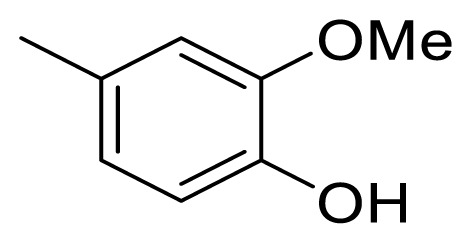 7.07 (dd, 1H, *J* = 8.4, 2.0) 7.03 (d, 1H, *J* = 2.0) 6.92 (d, 1H, *J* = 8.4) 5.91 (two s, OH); 3.92 (s, OMe)
**5e**	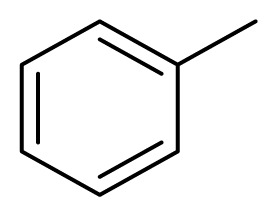 7.32-7.18 (m, 5H)	3.00-2.96 (A2B2, m 4H)	7.46 (d, 1H, *J* = 16.1)	6.60 (d, 1H, *J* = 16.1)	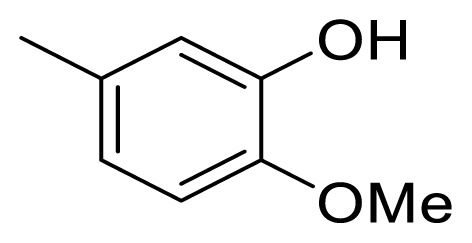 7.14 (d, 1H, *J* = 2.0) 7.04 (dd, 1H, *J* = 8.4, 2.0) 6.84 (d, 1H, *J* = 8.4), 3.92 (s, OMe) 5.72 (s, OH)
**5f**	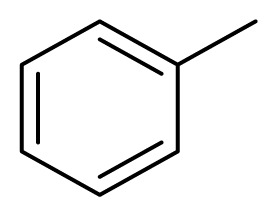 7.32-7.24 (m, 4H) 7.17-7.14 (m, 1H)	3.00-2.90 (A2B2, m, 4H)	7.51 (d, 1H, *J* = 16.1)	6.64 (d, 1H, *J* = 16.1)	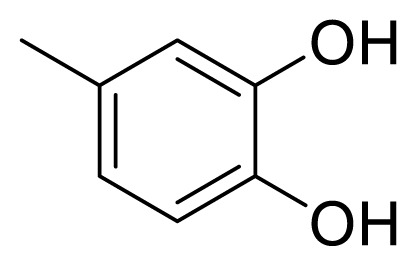 8.46 (bs, OH),8.17 (bs, OH) 7.18 (d, 1H, *J* = 2.0) 7.07 (dd, 1H, *J* = 8.2, 2.0) 6.87 (d,1H, *J* = 8.2)
**5g**	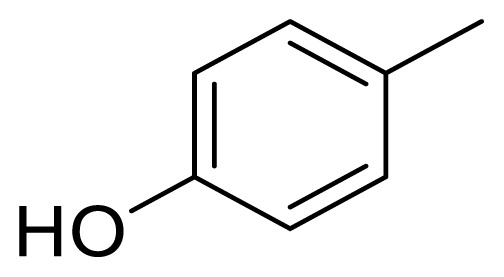 7.09 (dm, 2H, *J* = 8.4) 6.77 (dm, 2H, *J* = 8.4)	3.00-2.94 (A_2_B_2_, m, 4H)	7.54 (d, 1H, *J* = 16.4)	6.73 (d, 1H, *J* = 16.4)	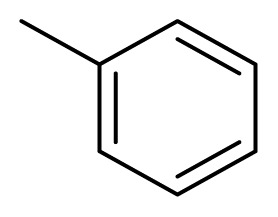 7.53-7.51 (m, 2H) 7.41-7.38 (m, 3H)
**5h**	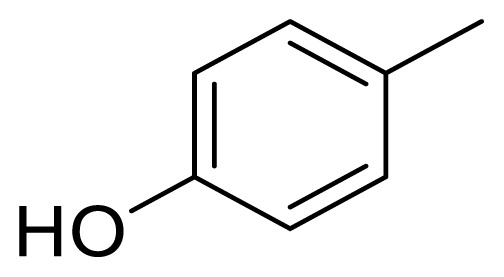 8.84 (s, OH), 8.04(s,OH), 7.08 (dm, 2H, *J* = 8.4) 6.74 (dm, 2H, *J* = 8.4)	2.94-2.90 (A_2_B_2,_ m,_,_4H)	7.56 (d, 1H, *J* = 16.1)	6.68 (d, 1H, *J* = 16.1)	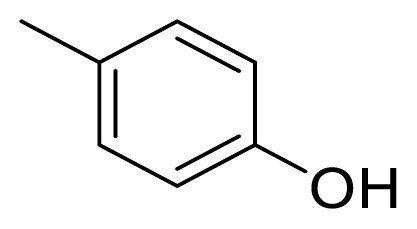 7.55 (dm, 2H, *J* = 8.4) 6.88 (dm, 2H, *J* = 8.4)
**5i**	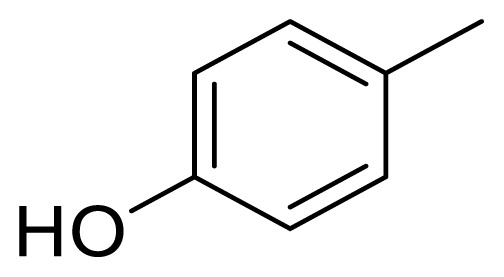 8.07 (OH), 7.08 (dm, 2H, *J* = 8.8) 6.74 (dm, 2H, *J* = 8.8)	2.96-2.82 (A_2_B_2,,_ m, 4H)	7.58 (d, 1H, *J* = 16.2)	6.72 (d, 1H, *J* = 16.2)	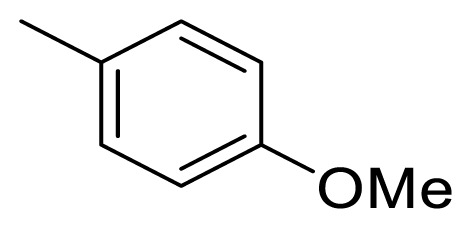 7.63 (dm, 2H, *J* = 8.8) 6.98 (dm, 2H, *J* = 8.8), 3.84 (s, OMe)
**5j**	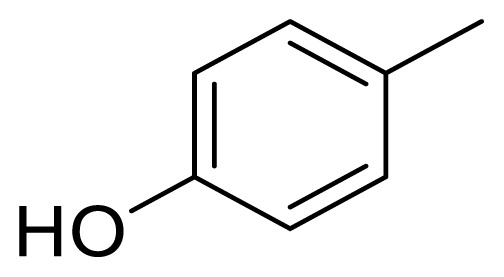 8.09 (OH), 7.08 (dm, 2H, *J* = 8.4) 6.75 (dm, 2H, *J* = 8.4)	A_2_ part 2.94-2.82 (A_2_B_2,_ m, 4H,)	7.54 (d, 1H, *J* = 16.2)	6.71 (d, 1H, *J* = 16.2)	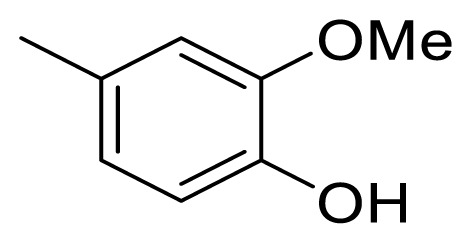 8.03 (OH) 7.31 (d, 1H, *J* = 1.9) 7.16 (dd, 1H, *J* = 8.2, 1.9) 6.87 (d, 1H, *J* = 8.2), 3.90 (s, OMe)
**5k**	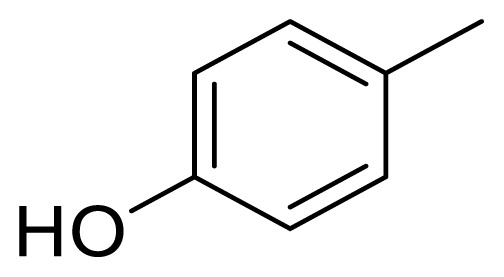 8.05(OH), 7.09 (dm, 2H, *J* = 8.4) 6.75 (dm, 2H, *J* = 8.4)	2.95-2.82 (A_2_ B_2,,_ m, 4H)	7.51 (d, 1H, *J* = 16.2)	6.69 (d, 1H, *J* = 16.2)	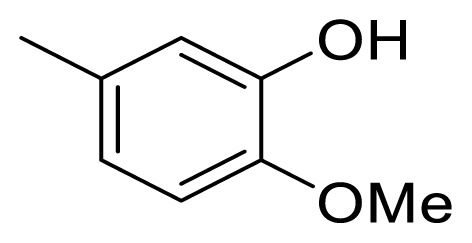 7.79 (OH), 7.19(d, 1H, *J* = 2.0) 7.13 (dd, 1H, *J* = 8.3, 2.0) 6.99 (d, 1H, *J* = 8.3), 3.88 (s, OMe)
**5l**	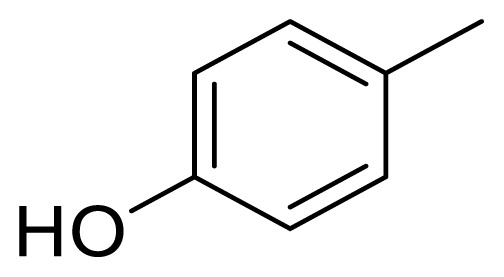 7.02 (dm, 2H, *J* = 8.4) 6.68 (dm, 2H, *J* = 8.4)	2.94--2.80 (A_2_B_2_, m, 4H)	7.46 (d, 1H, *J* = 16.1)	6.56 (d, 1H, *J* = 16.1)	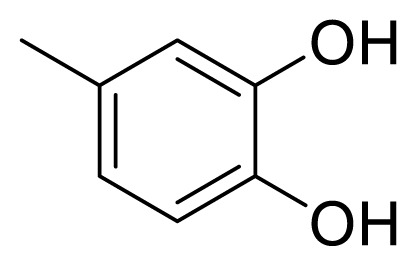 7.05 (d, 1H, *J* = 1.9) 6.95 (dd, 1H, *J* = 8.2, 1.9) 6.77 (d, 1H, *J* = 8.2), 4.87 (bs, 2 x OH)

**Table 2:** ^13^C-NMR data of **5a–5l** (**d** (ppm); J (Hz)).

Compound	Ar^2^	C(5)	C-(4)	C-(3)	C-(2)	C-(1)	Ar^1^
**5a**	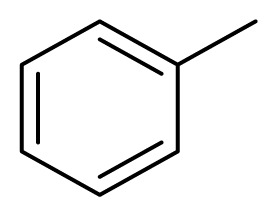 141.5 (C-1″) 128.6^a^ (C-2″/6″) 128.5 a (C-3″/5″) 126.4 (C-4″)	30.4	42.7	199.6	126.4	142.9	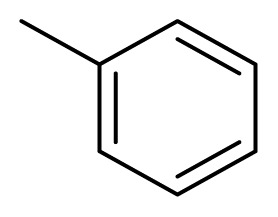 134.7 (C-1′) 129.2^a^ (C-2′/6′) 128.8^a^ (C-3′/5′) 130.7 (C-4′)
**5b**	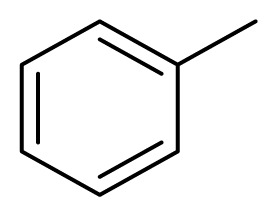 141.4 (C-1″) 128.8 a (C-2″/6″) 128.6 a (C-3″/5″) 126.4 (C-4″)	30.6	42.5	200.5	123.9	143.5	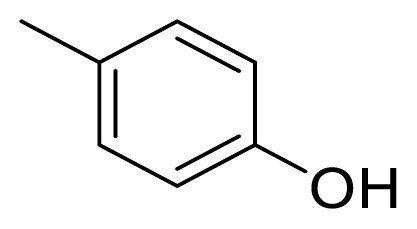 127.1 (C-1′) 130.6 (C-2′/6′) 116.3(C-3′/5′) 158.6 (C-4′)
**5c**	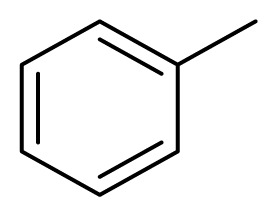 141.6 (C-1″) 128.7 a (C-2″/6″) 128.6 a (C-3″/5″) 126.3 (C-4″)	30.5	42.6	199.5	124.2	142.7	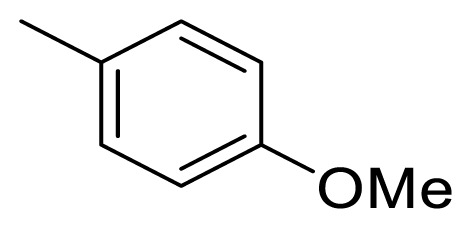 127.3 (C-1′) 130.2 (C-2′/6′) 114.6 (C-3′/5′) 161.8 (C-4′), 55.6 (OMe)
**5d**	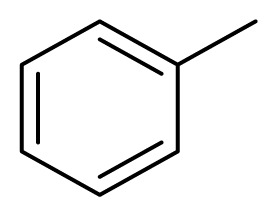 141.5 (C-1″) 128.7 a (C-2″6″) 128.6 a (C-3″/5″) 126.3 (C-4″)	30.5	42.4	199.5	123.7	143.2	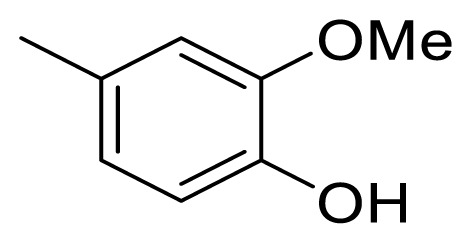 127.2 (C-1′), 109.6 (C-2′) 148.4 (C-3′), 147.0 (C-4′) 115.0 (C-5′), 124.2 (C-6′), 56.2 (OMe)
**5e**	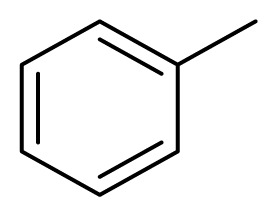 141.5 (C-1″) 128.7 a (C-2″/6″) 128.6 a (C-3″/5″) 126.3 (C-4″)	30.5	42.7	199.5	122.4	142.9	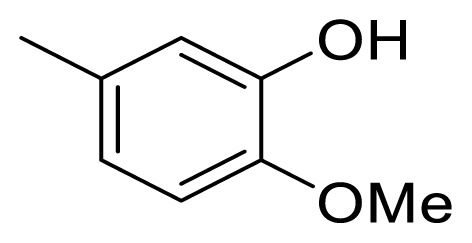 128.3 (C-1′), 110.8 (C-2′) 149.0 (C-3′), 146.1 (C-4′) 113.3 (C-5′), 124.7 (C-6′), 56.2 (OMe)
**5f**	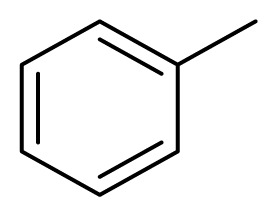 142.0 (C-1″) 128.6 a (C-2″/6″) 128.5 a (C-3″/5″) 126.0 (C-4″)	30.2	41.9	198.3	123.8	142.7	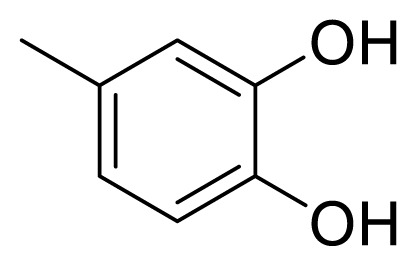 127.3 (C-1′), 114.6 (C-2′) 148.0 (C-3′), 145.6 (C-4′) 115.7 (C-5′), 122.1 (C-6′)
**5g**	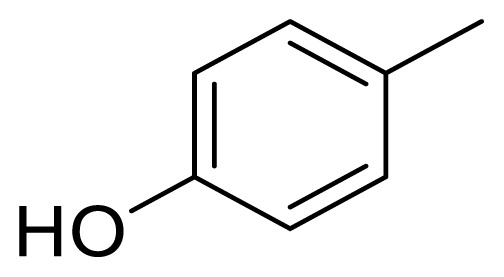 133.3 (C-1″) 129.7 (C-2″/6″) 115.5 (C-3″/5″) 154.3 (C-4″)	29.6	42.9	200.3	126.2	143.2	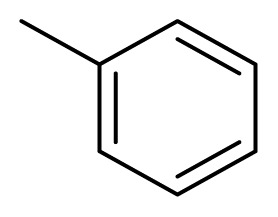 134.7 (C-1′) 129.2 a (C-2′/6′) 128.5 a (C-3′/5′) 130.7 (C-4′)
**5h**	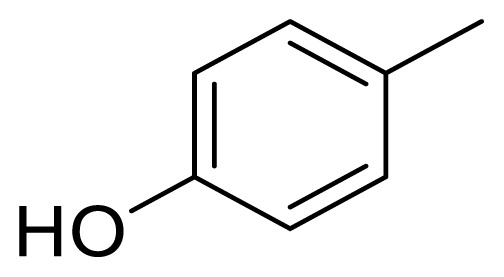 132.5 (C-1″) 129.3^a^ (C-2″/6″) 115.2^b^ (C-3″/5″) 155.8 (C-4″)	29.4	42.4	198.6	123.7	142.2	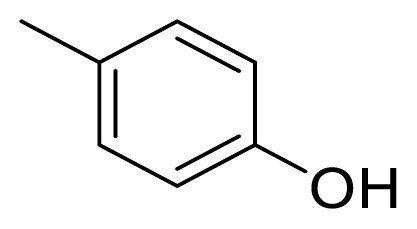 126.7 (C-1′) 130.2^a^ (C-2′/6′) 116.2^b^ (C-3′/5′) 159.9 (C-4′)
**5i**	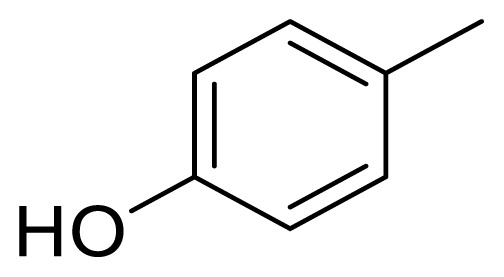 132.4 (C-1″) 129.3^a^ (C-2″/6″) 114.6^b^ (C-3″/5″) 155.7 (C-4″)	29.4	42.4	198.6	124.5	141.8	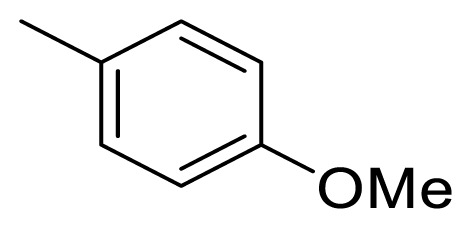 127.6 (C-1′) 130.2 a (C-2′/6′) 115.5 b (C-3′/5′) 161.9 (C-4′) 55.1 (OMe)
**5j**	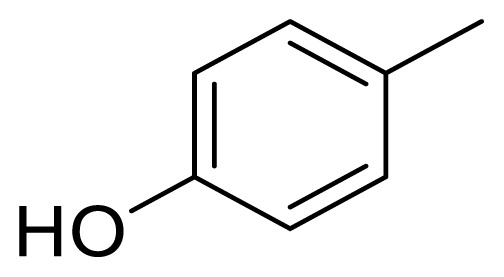 132.6 (C-1″) 129.5 (C-2″/6″) 115.2 (C-3″/5″) 155.8 (C-4″)	29.5	42.4	198.6	124.0	142.6	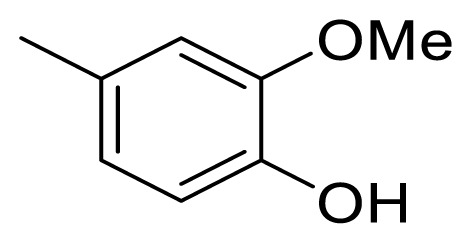 127.3 (C-1′), 110.8 (C-2′) 149.4 (C-3′), 148.1 (C-4′) 115.6 (C-5′), 123.4 (C-6′); 55.7 (OMe)
**5k**	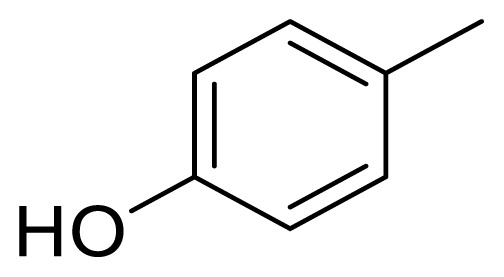 132.5 (C-1″) 129.5 (C-2″/6″) 113.9 (C-3″/5″) 155.7 (C-4″)	29.4	42.5	198.5	124.5	142.3	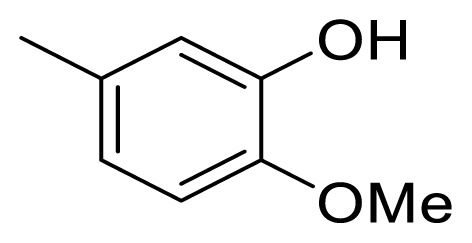 128.3 (C-1′), 111.6 (C-2′) 150.0 (C-3′), 147.0 (C-4′) 115.3 (C-5′), 121.8 (C-6′), 55.6 (OMe).
**5l**	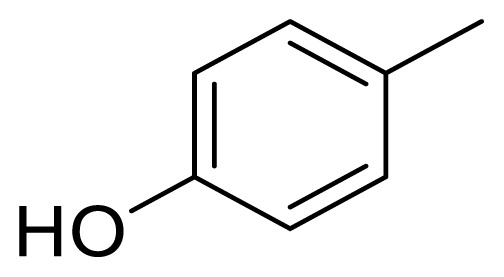 132.2 (C-1″) 129.2 (C-2″/6″) 115.0 (C-3″/5″) 155..4 (C-4″)	29.7	42.1	201.7	122.8	144.5	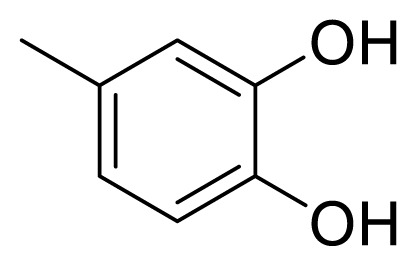 126.6 (C-1′), 114.2 (C-2′) 148.7 (C-3′), 145.7 (C-4′) 115.4 (C-5′), 122.4 (C-6′)

a,b Exchangeable carbons.

## Figures and Tables

**Table 1:** The IC_50_ values (in μM) of diarylpentanoids **5a**–**5l** against human cancer cells and healthy cells for 48 h.

Compound	U87-MG	MCF-7	PC-3	HPAEpiC
DOX control (+)	8.95	14.72	6.76	64.43
5a	107.49	11.38	50.15	84.77
5b	70.87	9.55	22.04	5.51
5c	213.37	50.21	438.75	92.26
5d	31.92	27.81	20.86	14.42
5e	133.37	21.82	95.82	26.35
5f	33.40	544.39	97.50	43.12
5g	127.74	16.17	290.88	21.13
5h	357.92	26.72	436.68	51.40
5i	233.33	5.92	9.74	14.91
5j	75.73	35.00	32.22	93.43
5k	52.56	202.72	45.12	69.33
5l	286.63	317.02	184.70	395.99

**Table 2:** SI values of diarylpentanoids **5a**–**5l** against several human cancer cell lines.

Compound	U87-MG cells	MCF-7 cells	PC-3 cells
DOX control (+)	7.20	4.38	9.53
5a	0.79	7.45	1.69
5b	0.08	0.58	0.25
5c	0.42	1.84	0.21
5d	0.45	0.52	0.69
5e	0.20	1.21	0.28
5f	1.29	0.08	0.44
5g	0.17	1.31	0.07
5h	0.14	1.92	0.12
5i	0.06	2.52	1.53
5j	1.23	2.67	2.90
5k	1.32	0.34	1.54
5l	1.38	1.25	2.14
