# Supplementary figures and images for: Synthesis of alnustone-like diarylpentanoids via a 4 + 1 strategy and assessment of their potential anticancer activity
Source: Turk J Chem. 2023 Oct 11;47(5):1249–59. doi: 10.55730/1300-0527.3609 (PMC10760875; doi:10.55730/1300-0527.3609)

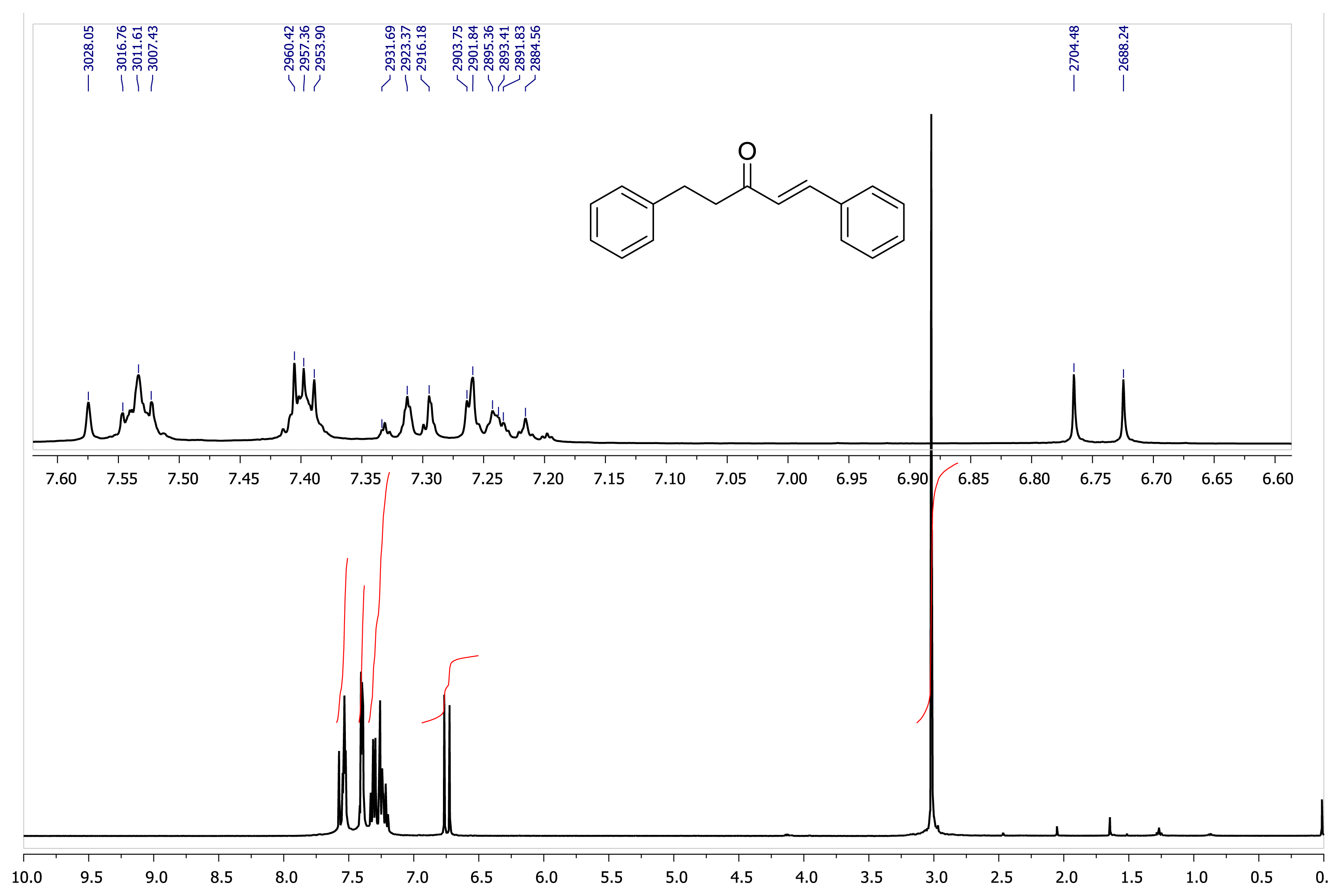

Supplement: Figure 1 — 1H-NMR spectrum of (E)-1,5-diphenylpent-1-en-3-one (5a) (CDCl3). [file turkjchem-47-5-1249s1.tif]

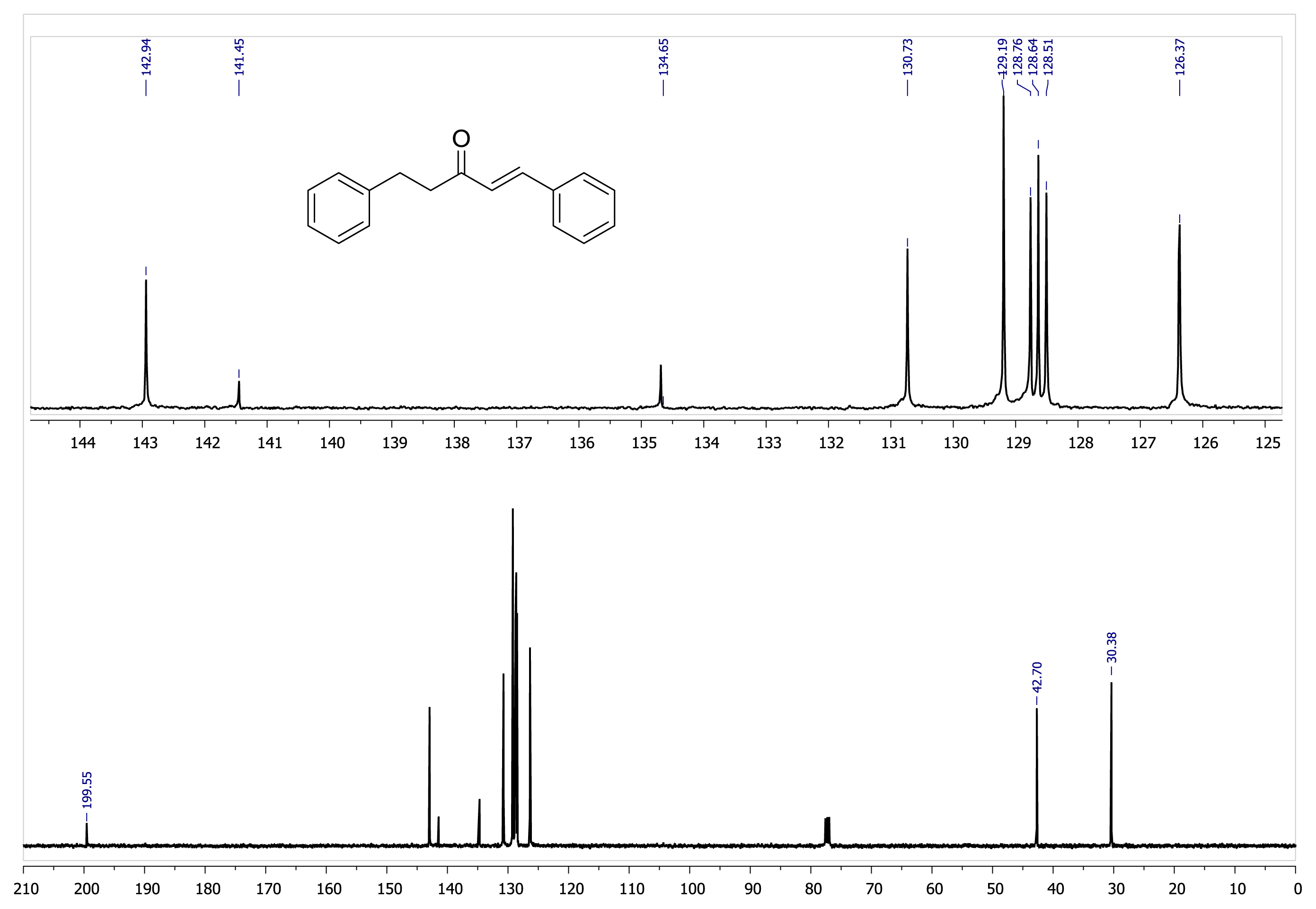

Supplement: Figure 2 — 13C-NMR spectrum of (E)-1,5-diphenylpent-1-en-3-one (5a) (CDCl3). [file turkjchem-47-5-1249s2.tif]

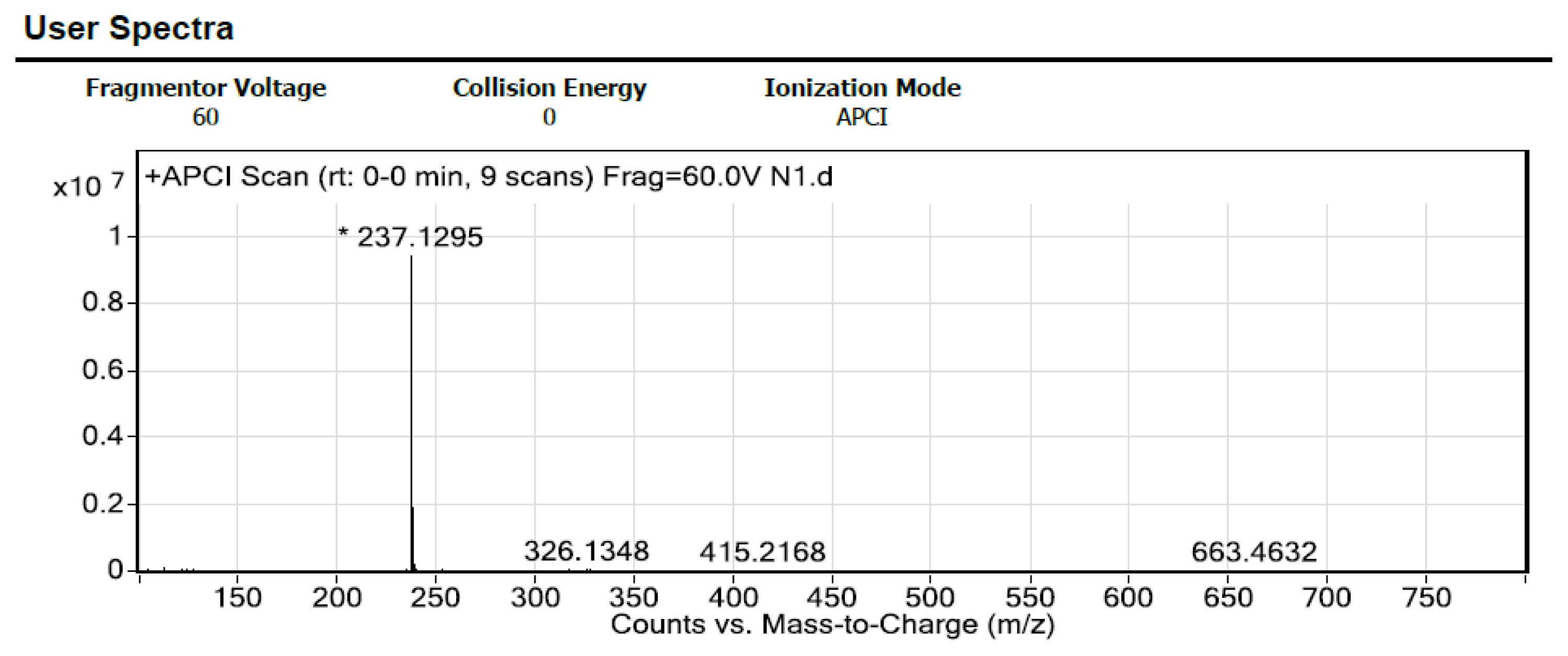

Supplement: Figure 3 — HRMS spectrum of (E)-1,5-diphenylpent-1-en-3-one (5a). (C17H16O+H)+, Calc: 237.1279. [file turkjchem-47-5-1249s3.tif]

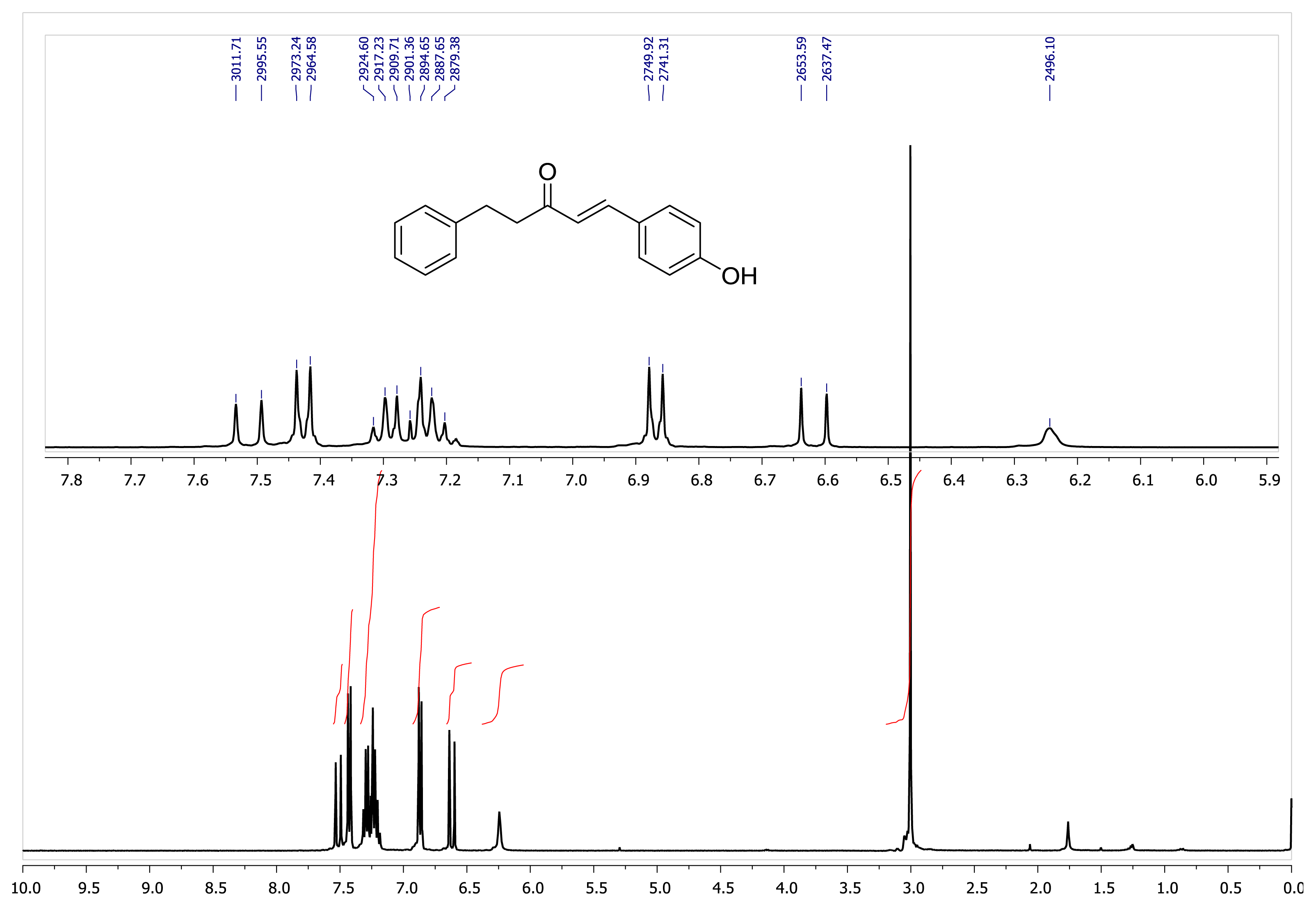

Supplement: Figure 4 — 1H-NMR spectrum of (E)-1-(4-hydroxyphenyl)-5-phenylpent-1-en-3-one (5b) (CDCl3). [file turkjchem-47-5-1249s4.tif]

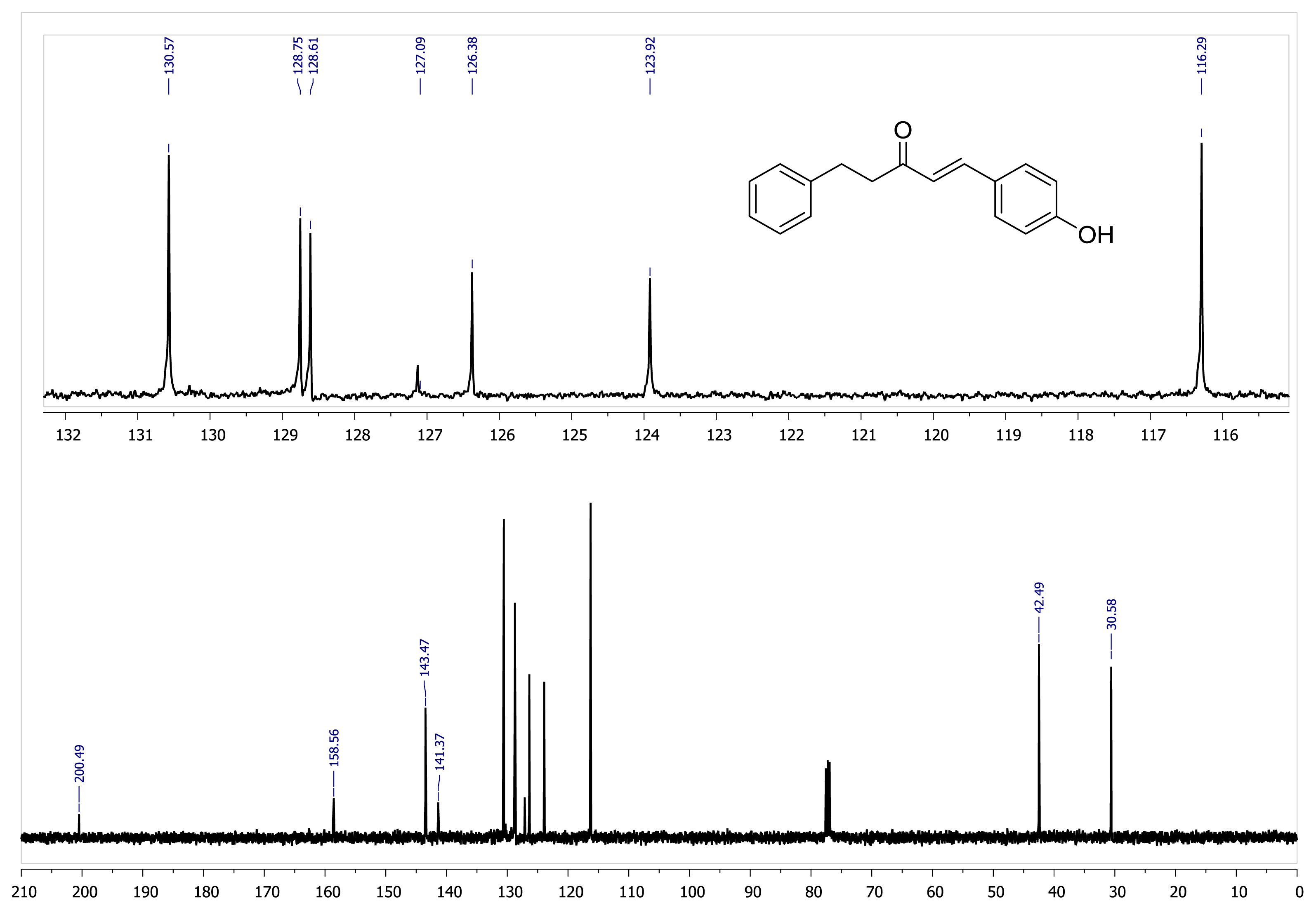

Supplement: Figure 5 — 13C-NMR spectrum of (E)-1-(4-hydroxyphenyl)-5-phenylpent-1-en-3-one (5b) (CDCl3). [file turkjchem-47-5-1249s5.tif]

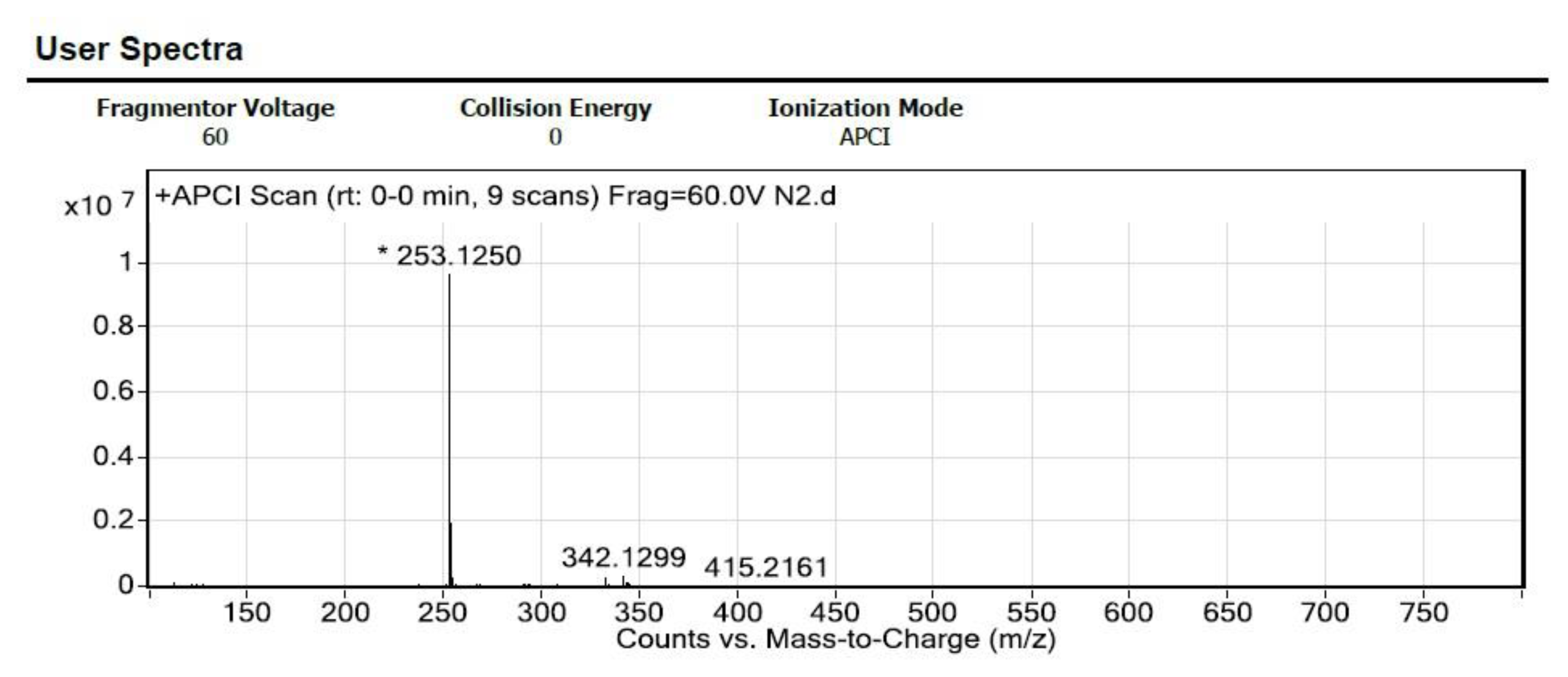

Supplement: Figure 6 — HRMS spectrum of (E)-1-(4-hydroxyphenyl)-5-phenylpent-1-en-3-one (5b). (C17H16O2+H)+, Calc: 253.1228. [file turkjchem-47-5-1249s6.tif]

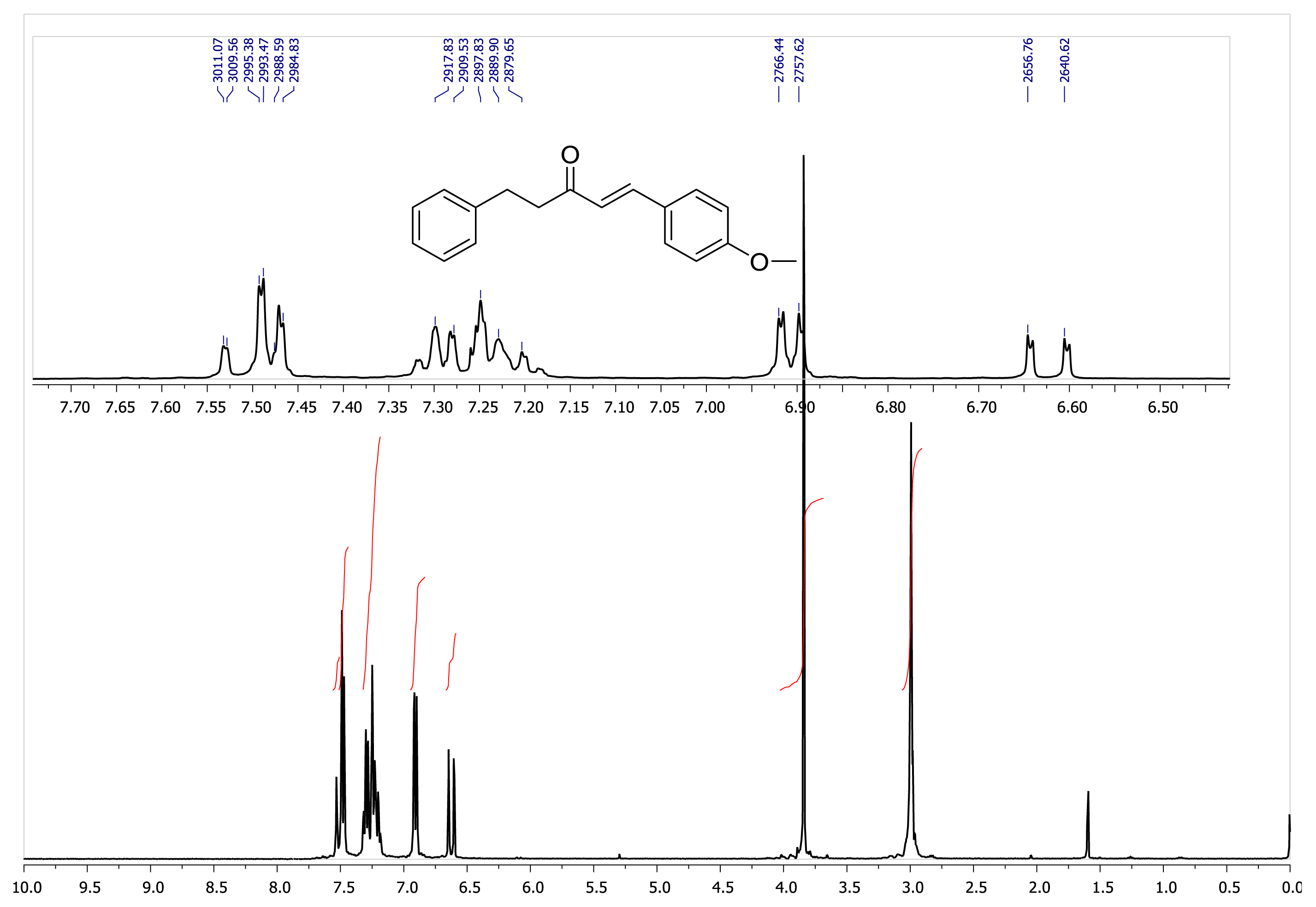

Supplement: Figure 7 — 1H-NMR spectrum of (E)-1-(4-methoxyphenyl)-5-phenylpent-1-en-3-one (5c) (CDCl3). [file turkjchem-47-5-1249s7.tif]

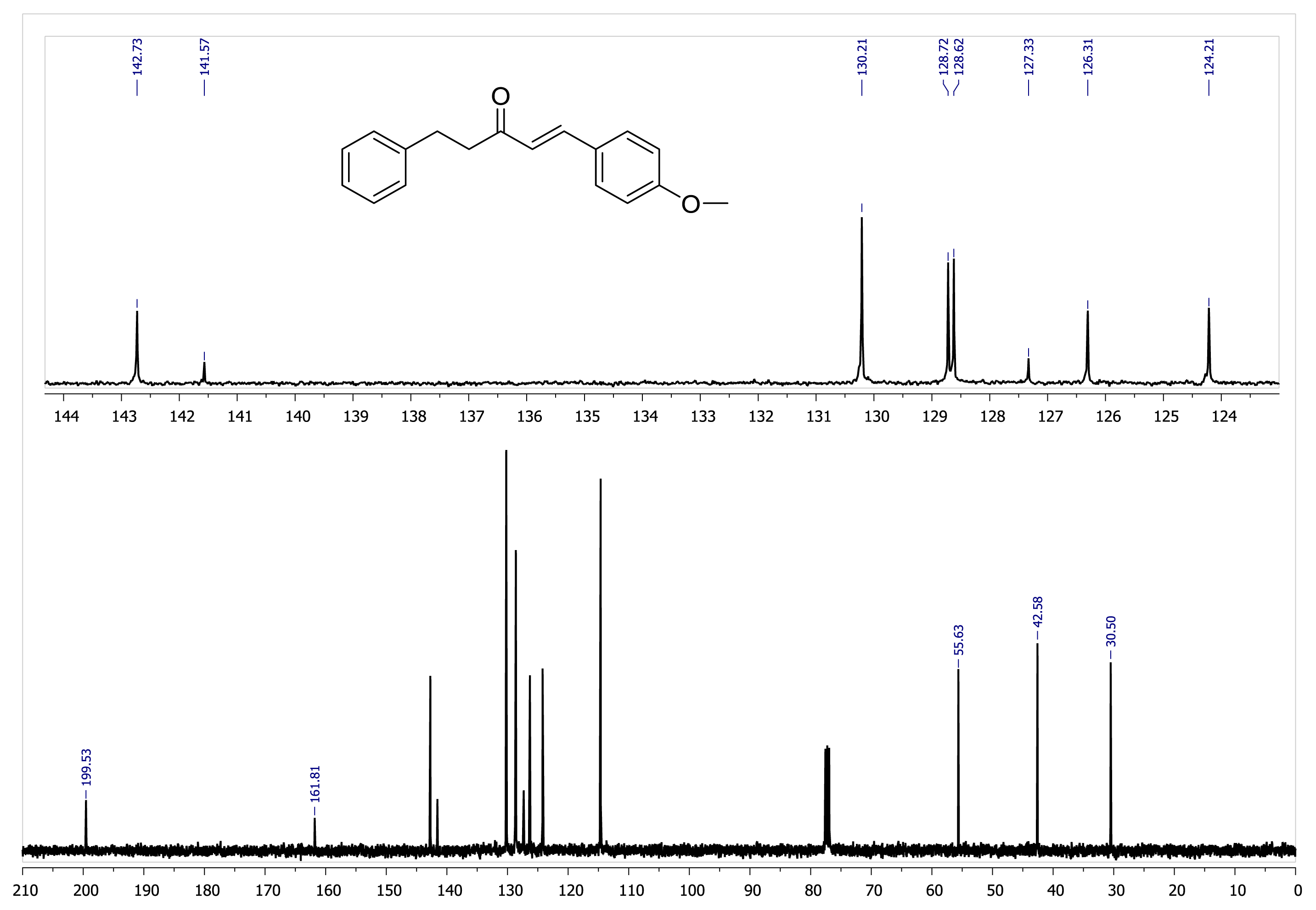

Supplement: Figure 8 — 13C-NMR spectrum of (E)-1-(4-methoxyphenyl)-5-phenylpent-1-en-3-one (5c) (CDCl3). [file turkjchem-47-5-1249s8.tif]

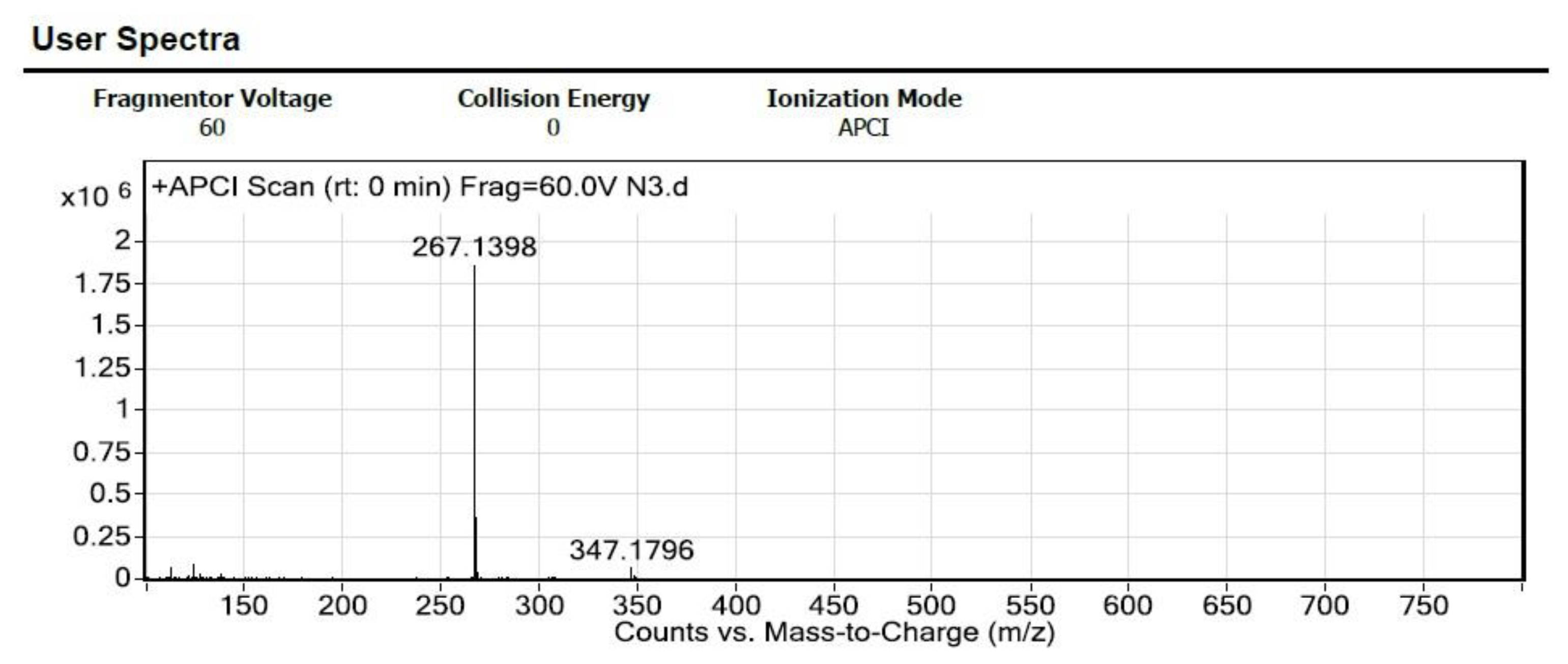

Supplement: Figure 9 — HRMS spectrum of (E)-1-(4-methoxyphenyl)-5-phenylpent-1-en-3-one (5c). (C18H18O2+H)+, Calc: 267.1385. [file turkjchem-47-5-1249s9.tif]

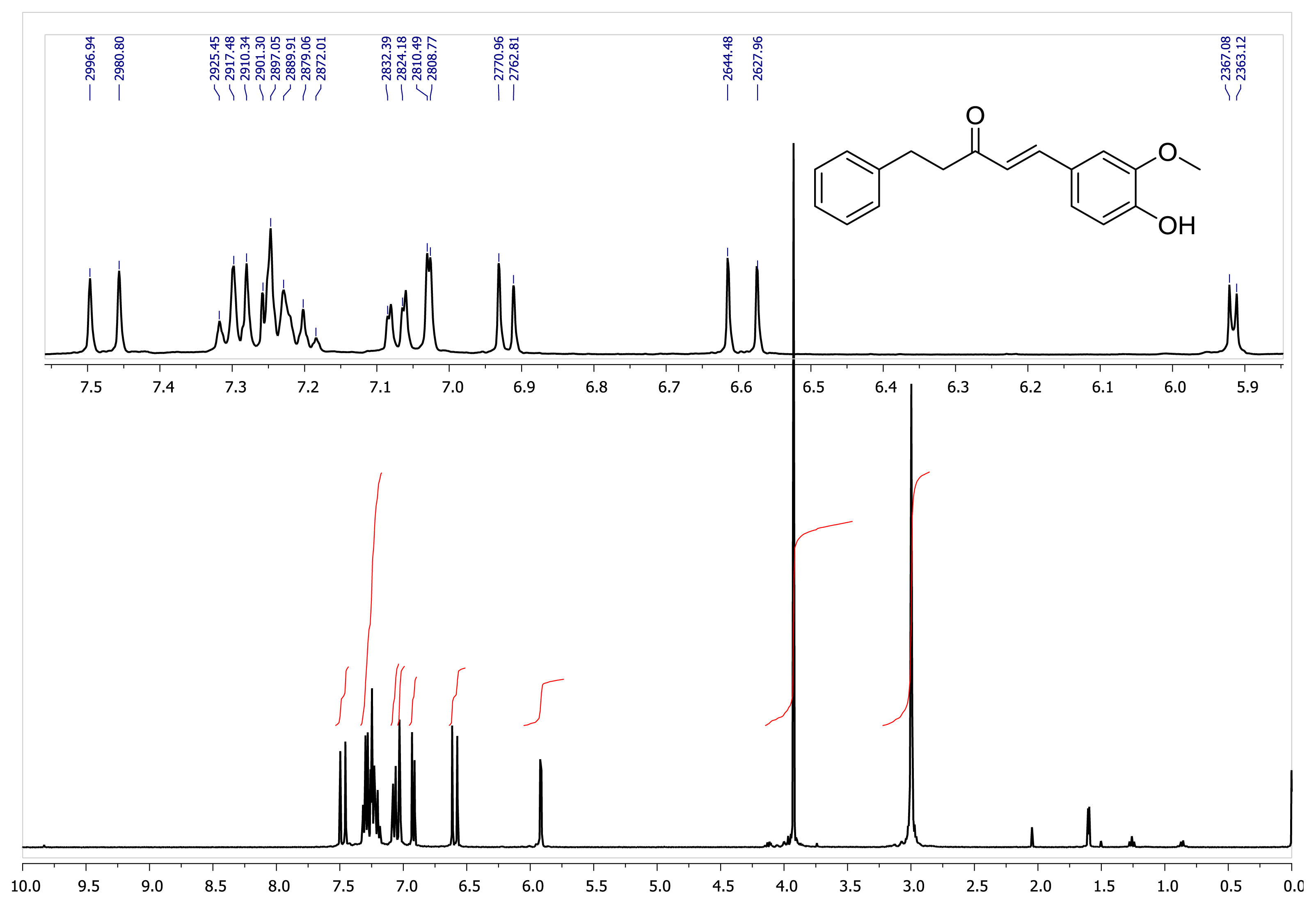

Supplement: Figure 10 — 1H-NMR spectrum of (E)-1-(4-hydroxy-3-methoxyphenyl)-5-phenylpent-1-en-3-one (5d) (CDCl3). [file turkjchem-47-5-1249s10.tif]

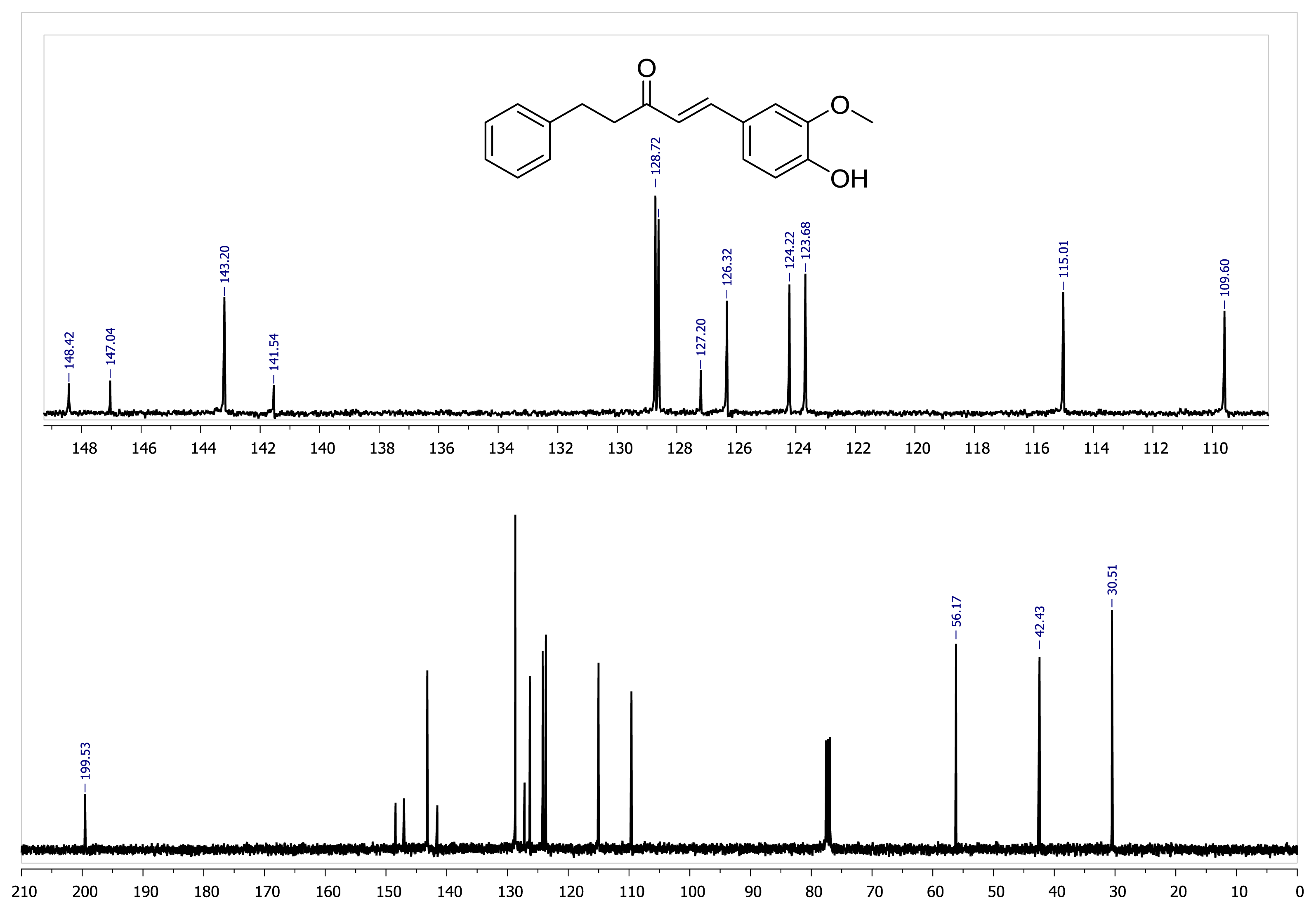

Supplement: Figure 11 — 13C-NMR spectrum of (E)-1-(4-hydroxy-3-methoxyphenyl)-5-phenylpent-1-en-3-one (5d) (CDCl3). [file turkjchem-47-5-1249s11.tif]

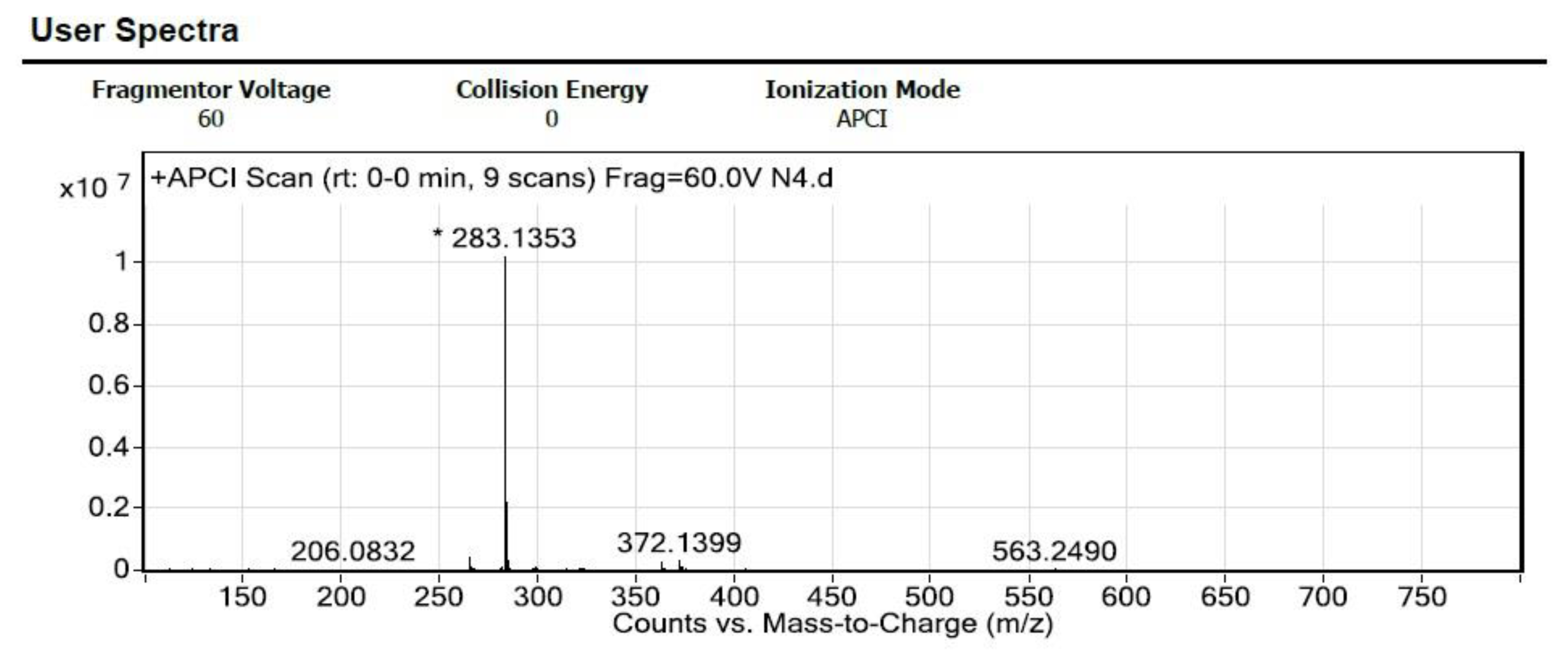

Supplement: Figure 12 — HRMS spectrum of (E)-1-(4-hydroxy-3-methoxyphenyl)-5-phenylpent-1-en-3-one (5d). (C18H18O3+H)+, Calc: 283.1334. [file turkjchem-47-5-1249s12.tif]

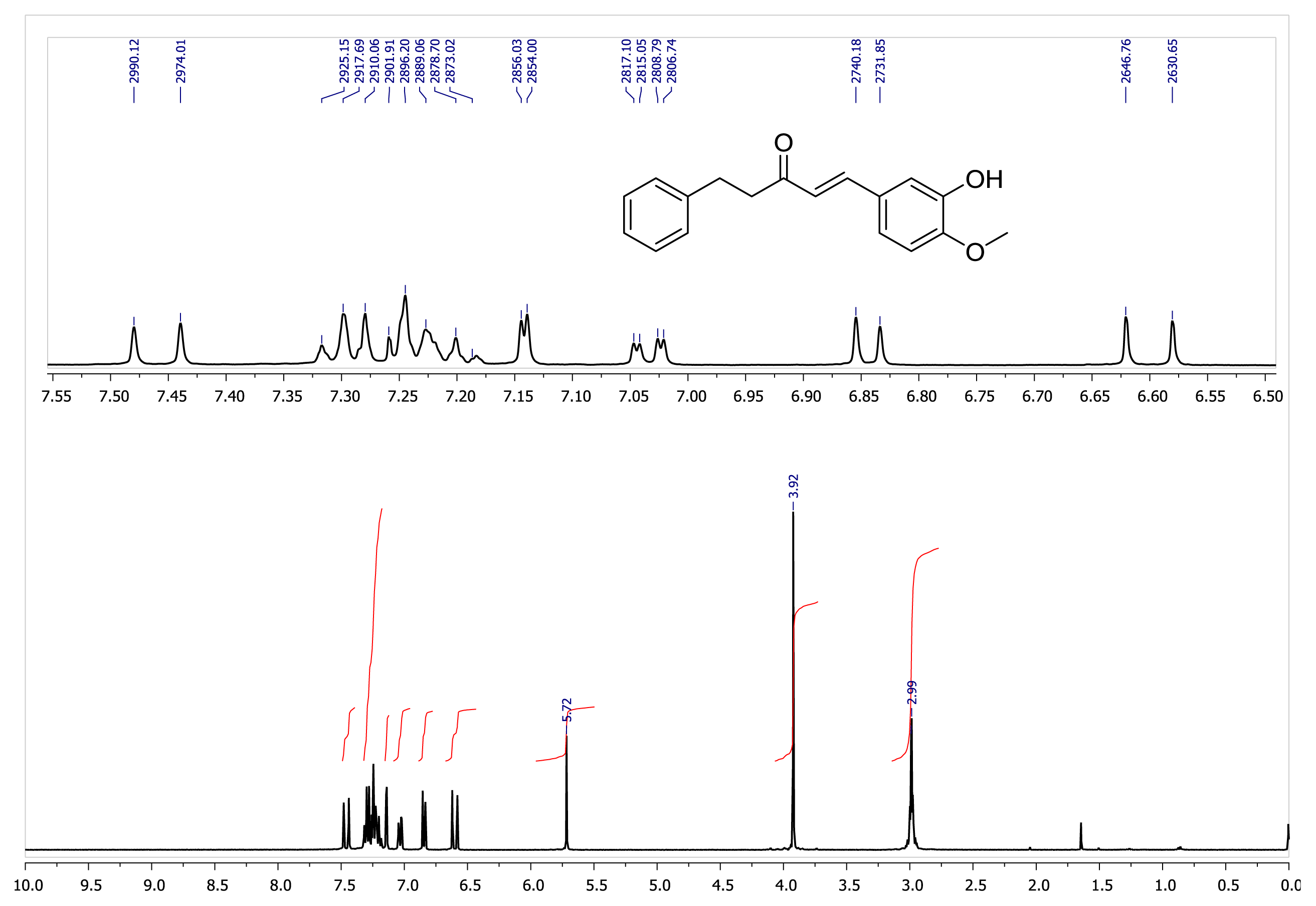

Supplement: Figure 13 — 1H-NMR spectrum of (E)-1-(3-hydroxy-4-methoxyphenyl)-5-phenylpent-1-en-3-one (5e) (CDCl3). [file turkjchem-47-5-1249s13.tif]

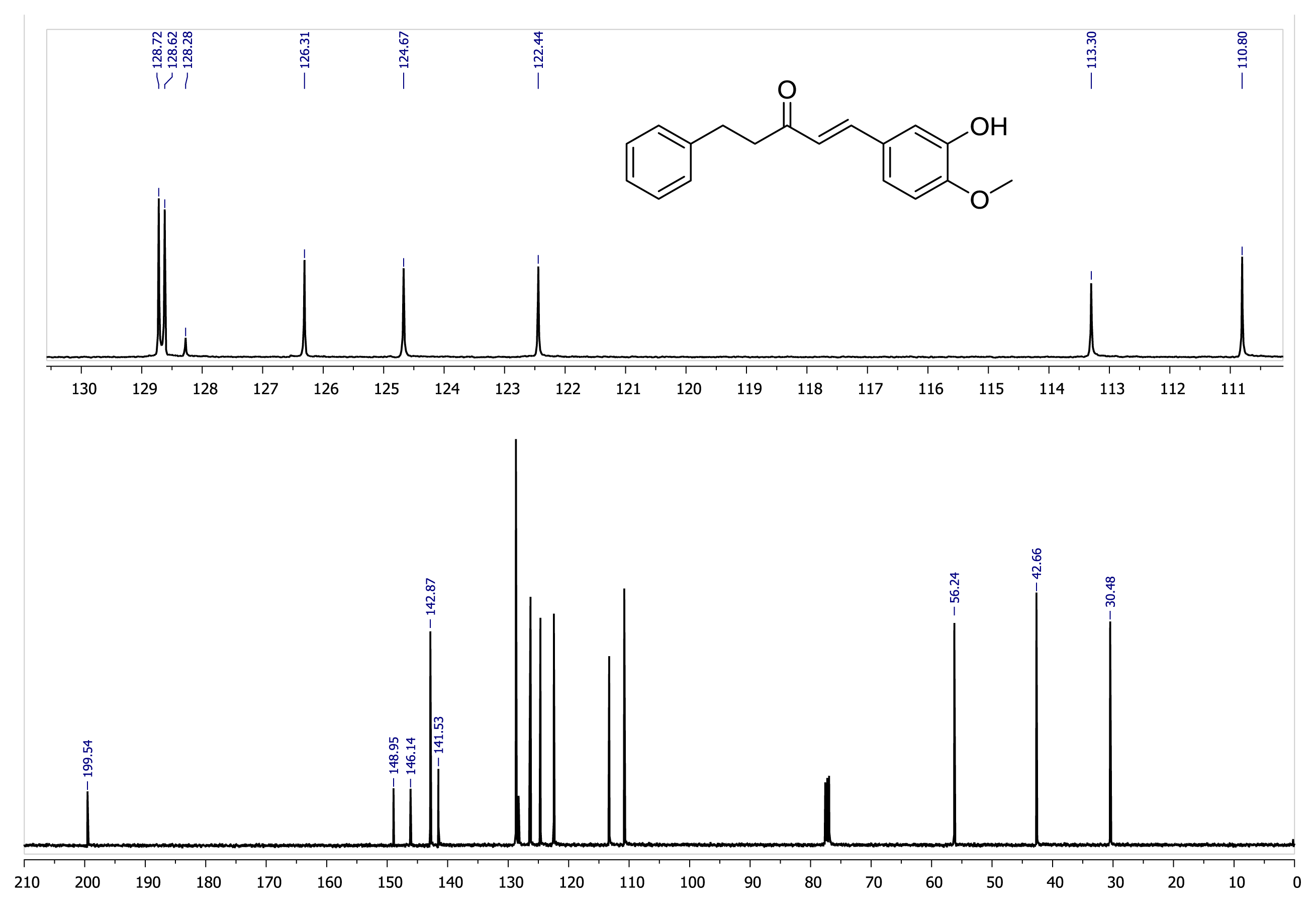

Supplement: Figure 14 — 13C-NMR spectrum of (E)-1-(3-hydroxy-4-methoxyphenyl)-5-phenylpent-1-en-3-one (5e) (CDCl3). [file turkjchem-47-5-1249s14.tif]

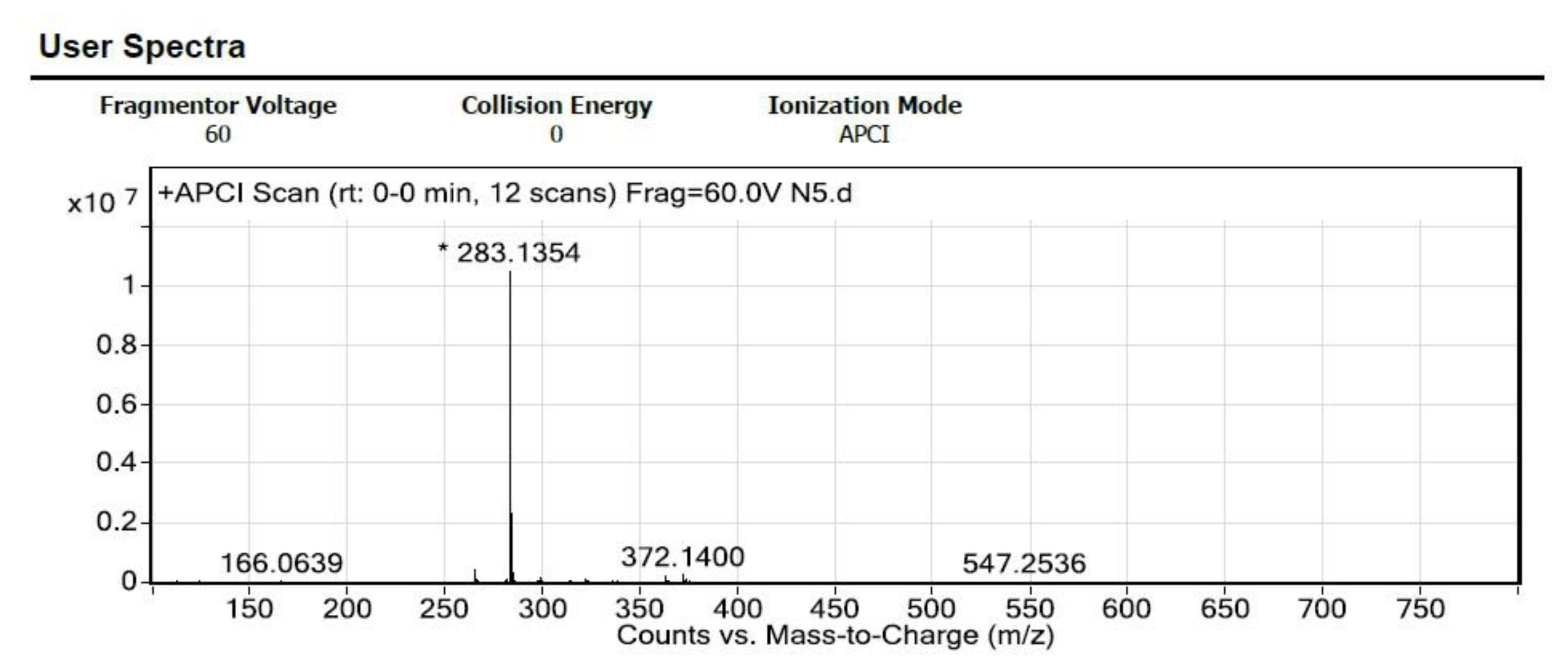

Supplement: Figure 15 — HRMS spectrum of (E)-1-(3-hydroxy-4-methoxyphenyl)-5-phenylpent-1-en-3-one (5e). (C18H18O3+H)+, Calc: 283.1334. [file turkjchem-47-5-1249s15.tif]

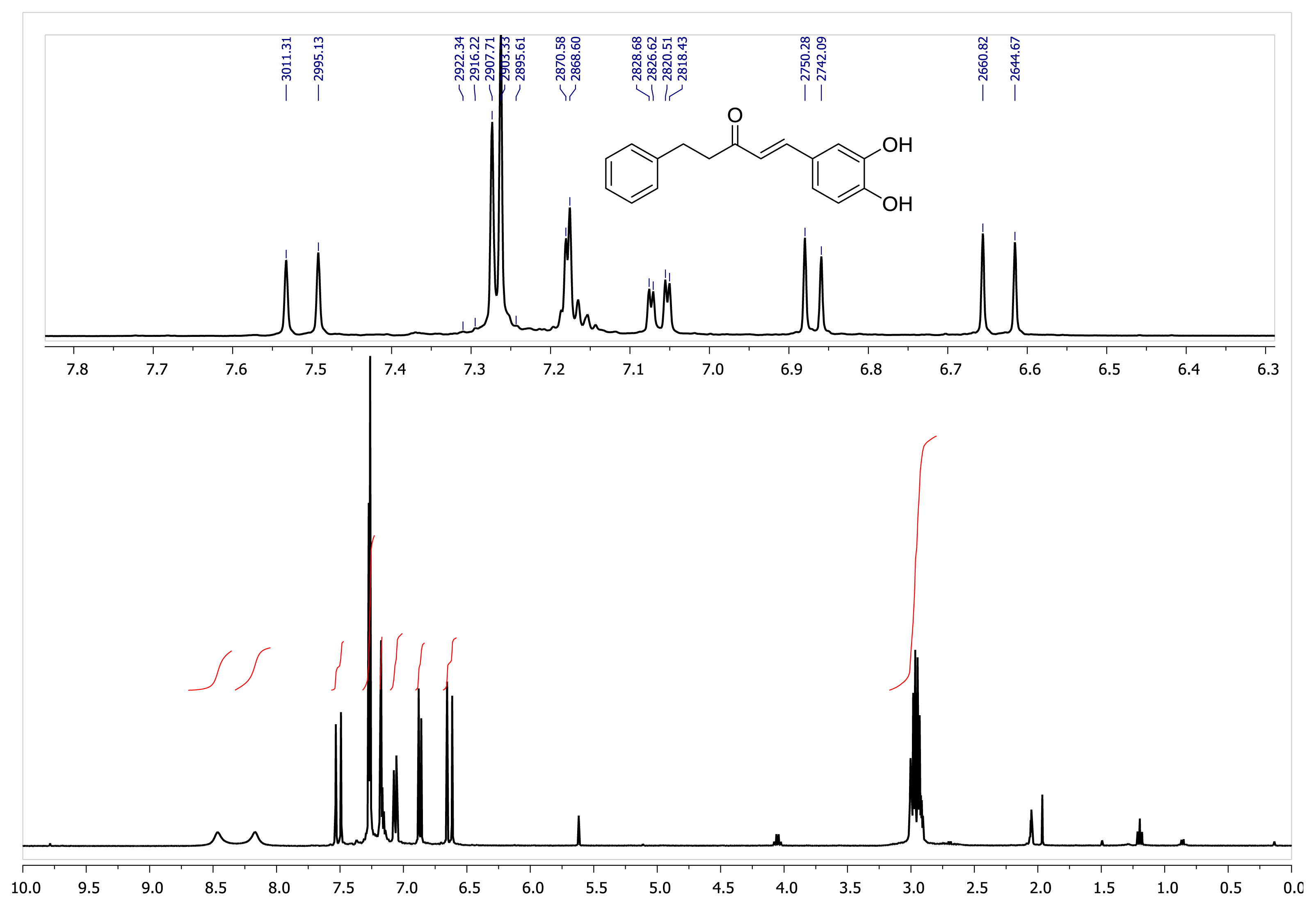

Supplement: Figure 16 — 1H-NMR spectrum of (E)-1-(3,4-dihydroxyphenyl)-5-phenylpent-1-en-3-one (5f) (Acetone-d6). [file turkjchem-47-5-1249s16.tif]

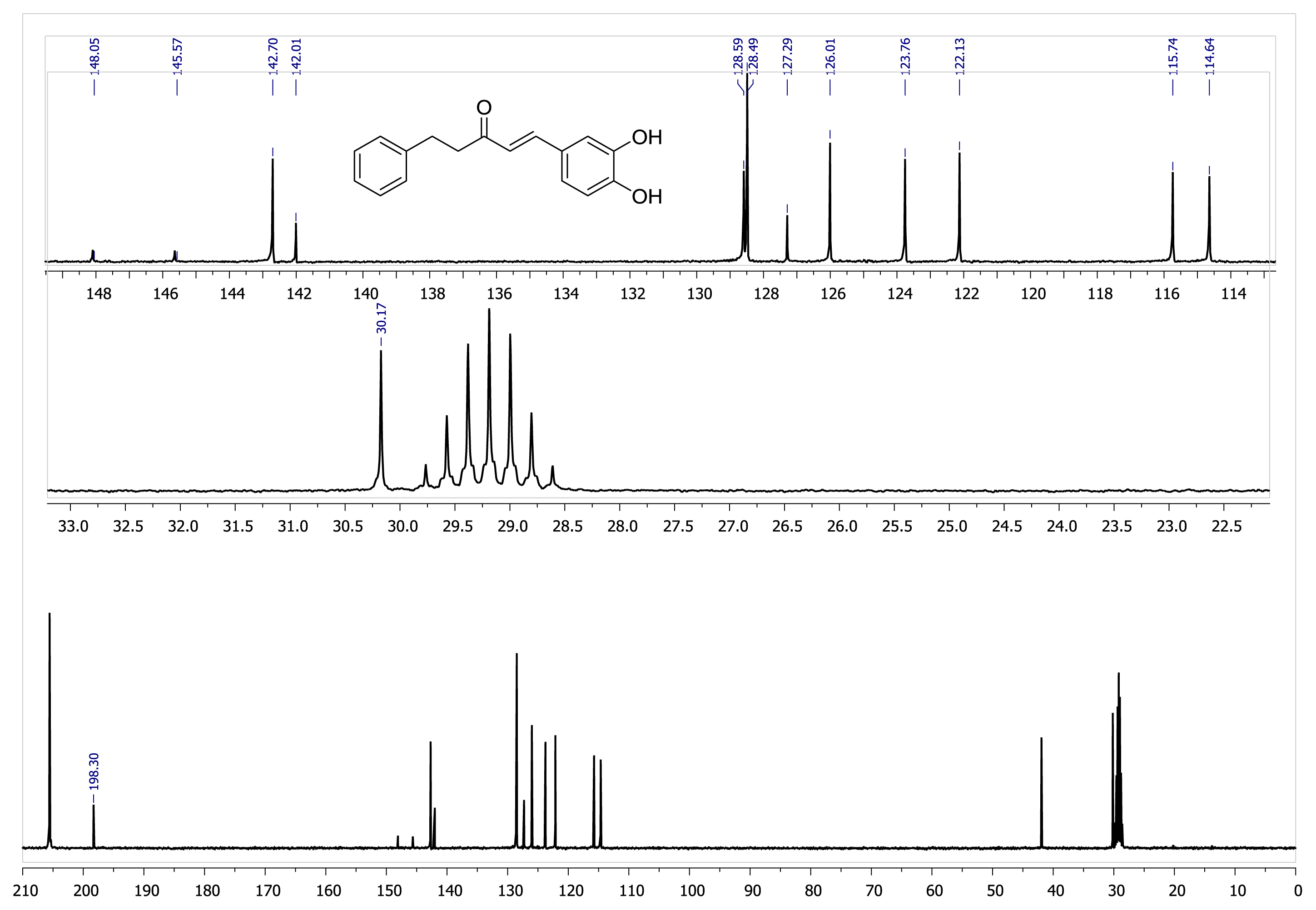

Supplement: Figure 17 — 13C-NMR spectrum of (E)-1-(3,4-dihydroxyphenyl)-5-phenylpent-1-en-3-one (5f) (Acetone-d6). [file turkjchem-47-5-1249s17.tif]

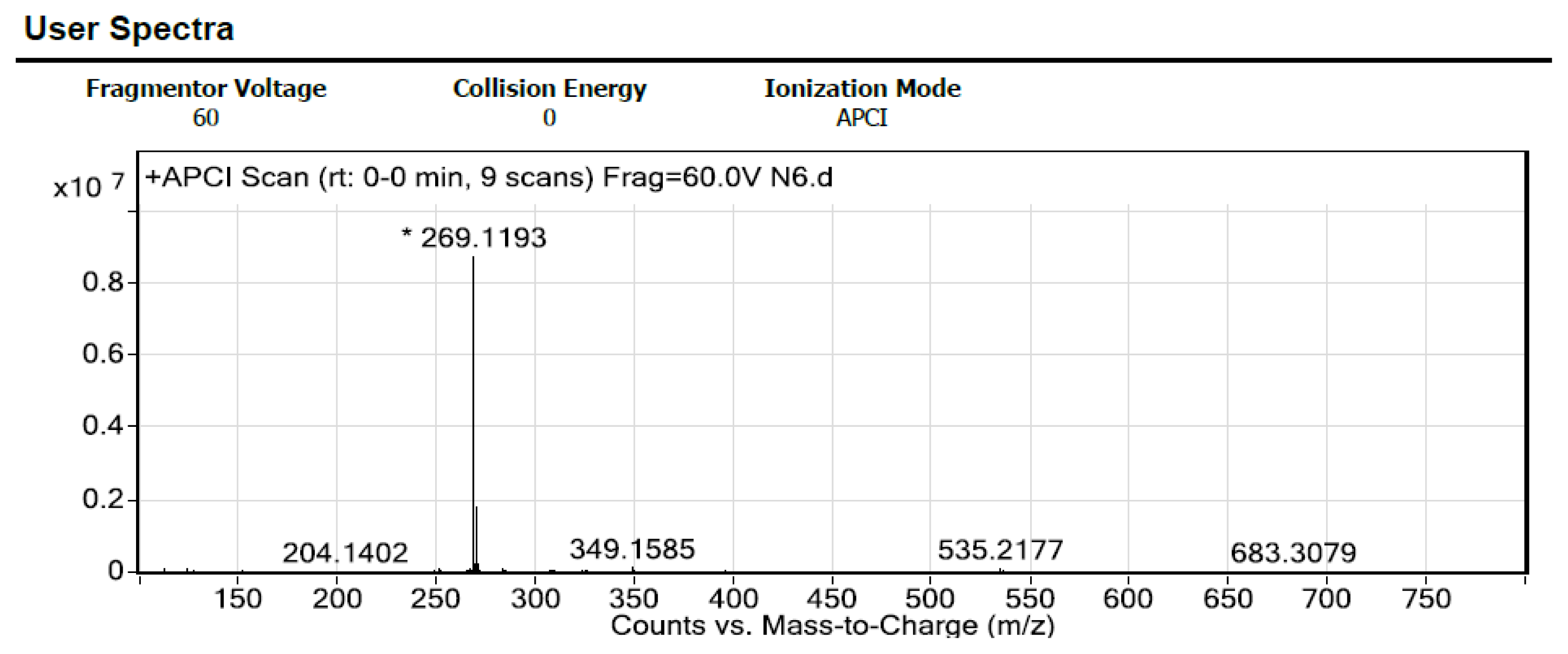

Supplement: Figure 18 — HRMS spectrum of (E)-1-(3,4-dihydroxyphenyl)-5-phenylpent-1-en-3-one (5f). (C17H16O3+H)+, Calc: 269.1177. [file turkjchem-47-5-1249s18.tif]

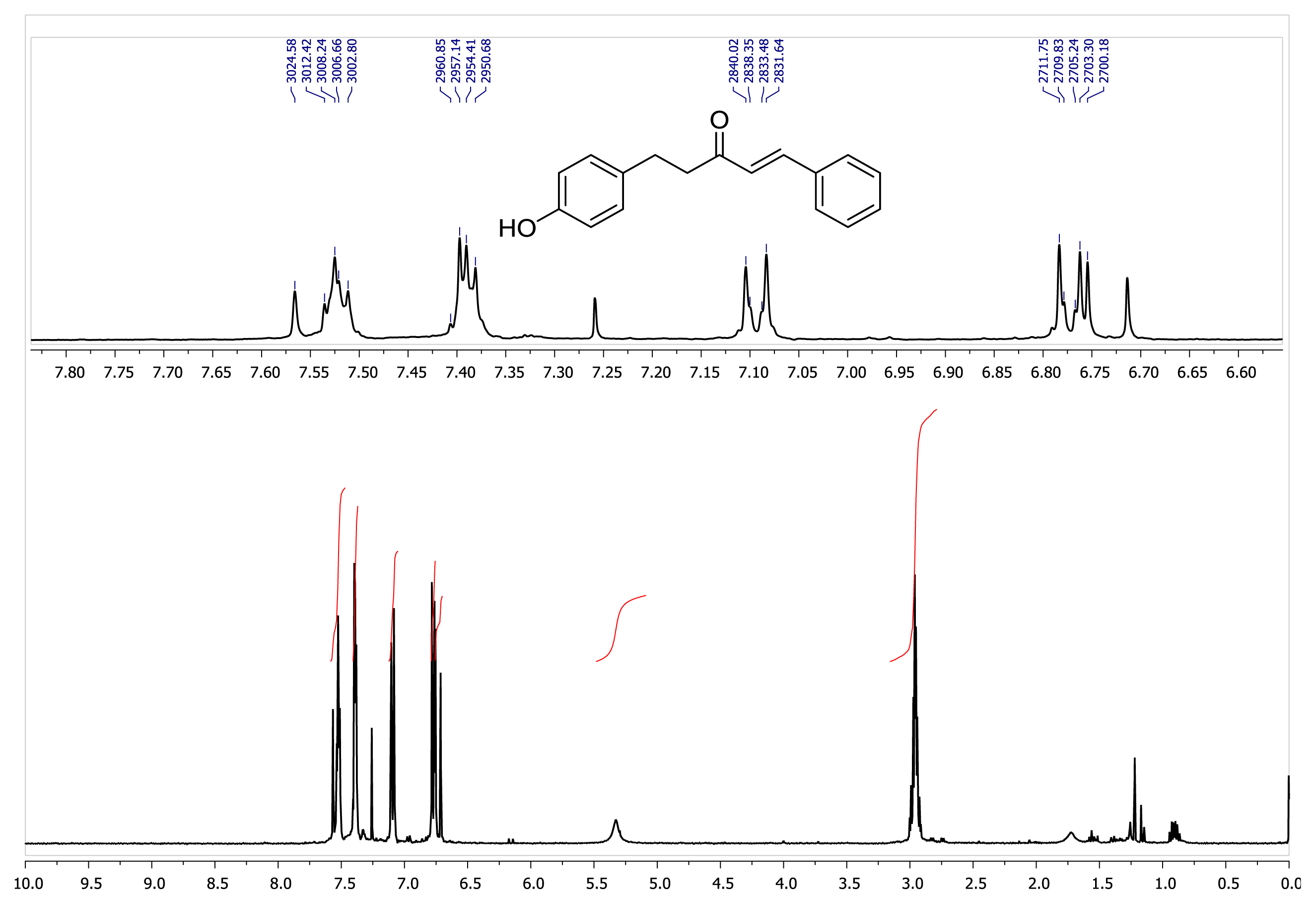

Supplement: Figure 19 — 1H-NMR spectrum of (E)-5-(4-hydroxyphenyl)-1-phenylpent-1-en-3-one (5g) (CDCl3). [file turkjchem-47-5-1249s19.tif]

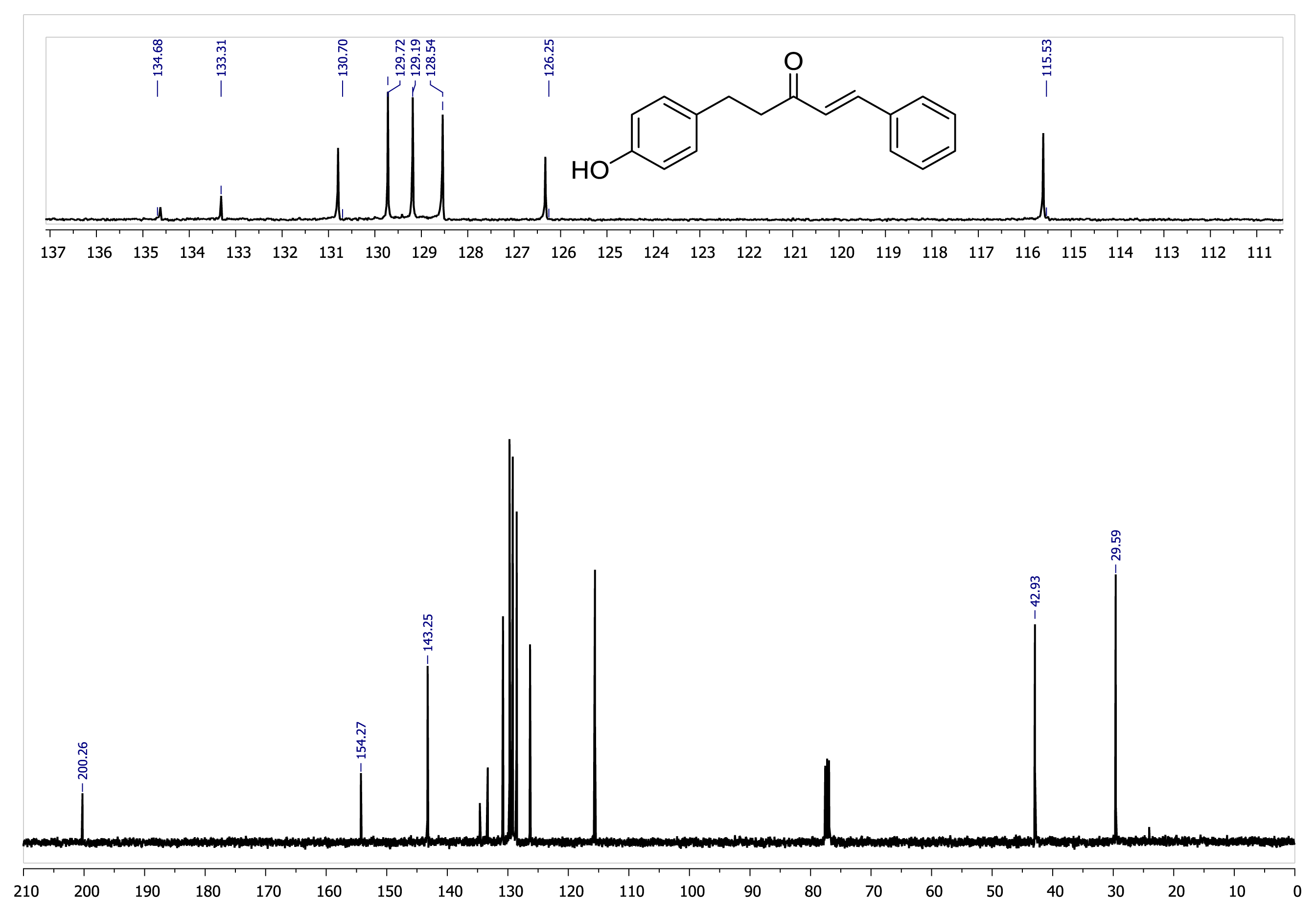

Supplement: Figure 20 — 13C-NMR spectrum of (E)-5-(4-hydroxyphenyl)-1-phenylpent-1-en-3-one (5g) (CDCl3). [file turkjchem-47-5-1249s20.tif]

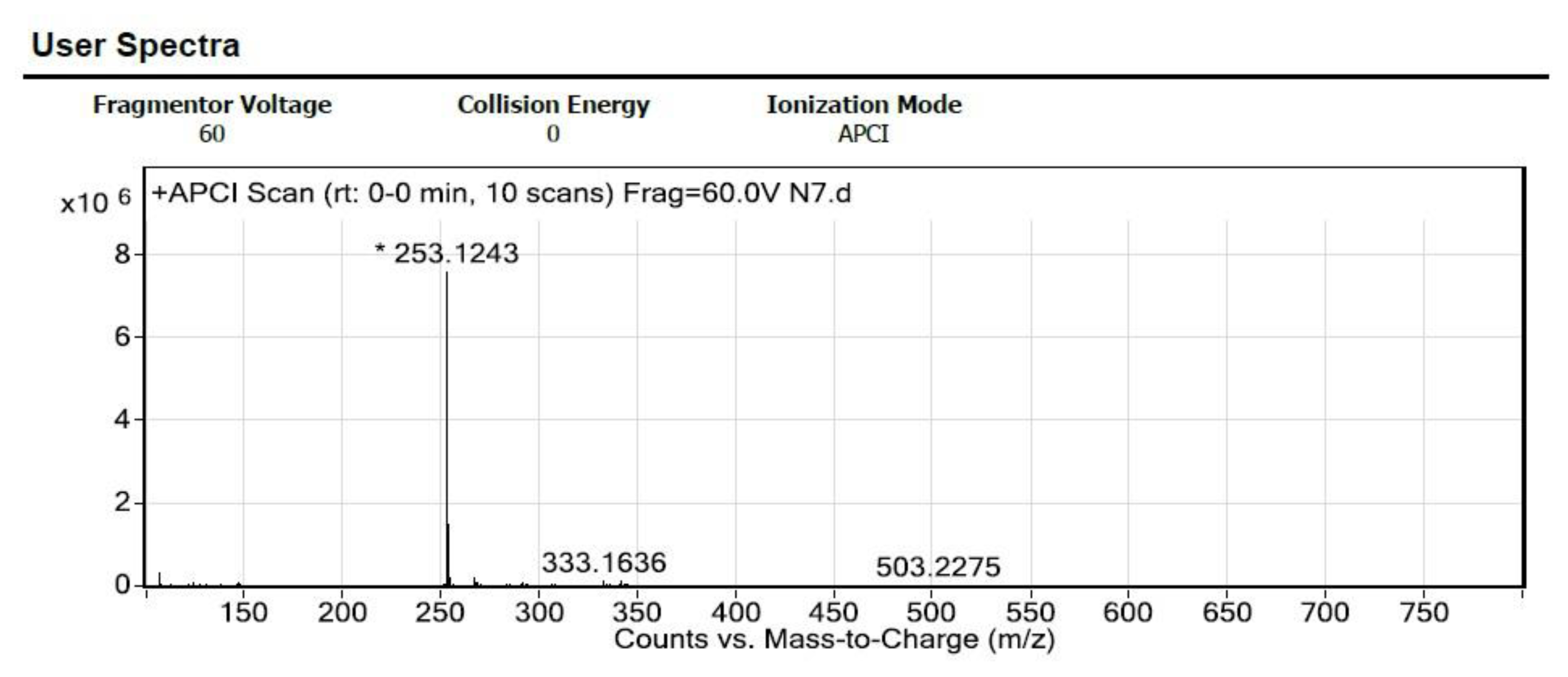

Supplement: Figure 21 — HRMS spectrum of (E)-5-(4-hydroxyphenyl)-1-phenylpent-1-en-3-one (5g). (C17H16O2+H)+, Calc: 253.1228. [file turkjchem-47-5-1249s21.tif]

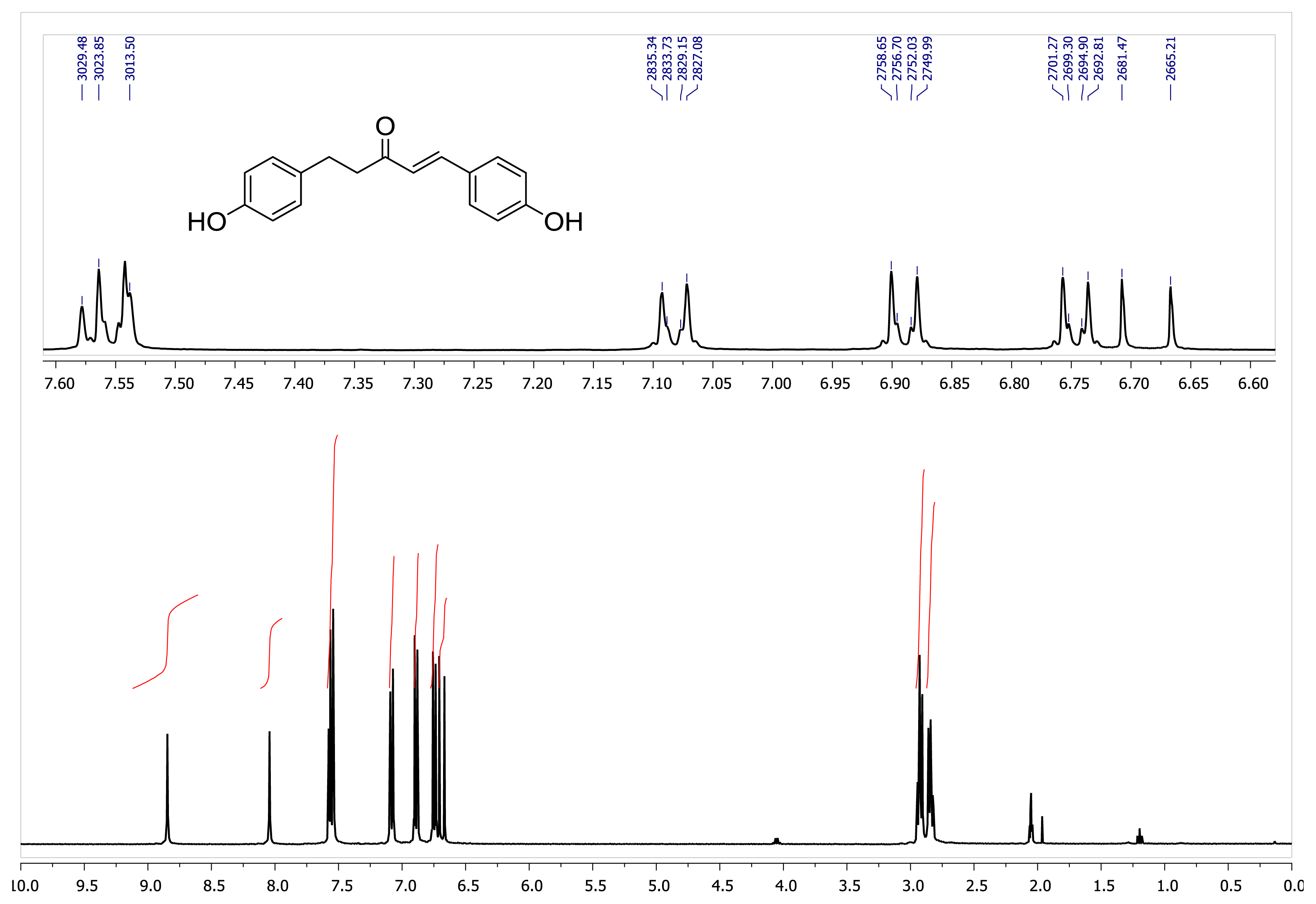

Supplement: Figure 22 — 1H-NMR spectrum of (E)-1,5-bis(4-hydroxyphenyl)pent-1-en-3-one (5h) (Acetone-d6). [file turkjchem-47-5-1249s22.tif]

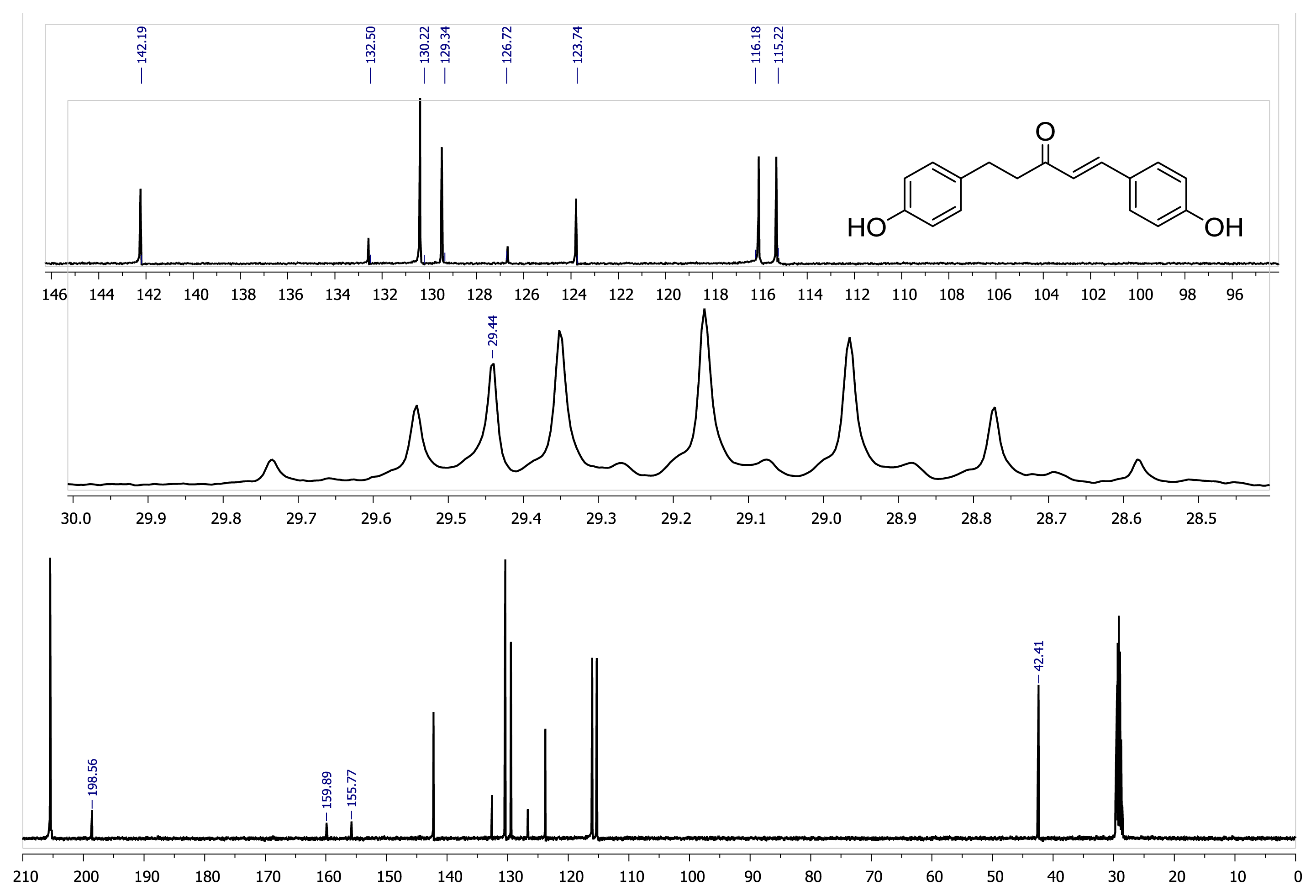

Supplement: Figure 23 — 13C-NMR spectrum of (E)-1,5-bis(4-hydroxyphenyl)pent-1-en-3-one (5h) (Acetone-d6). [file turkjchem-47-5-1249s23.tif]

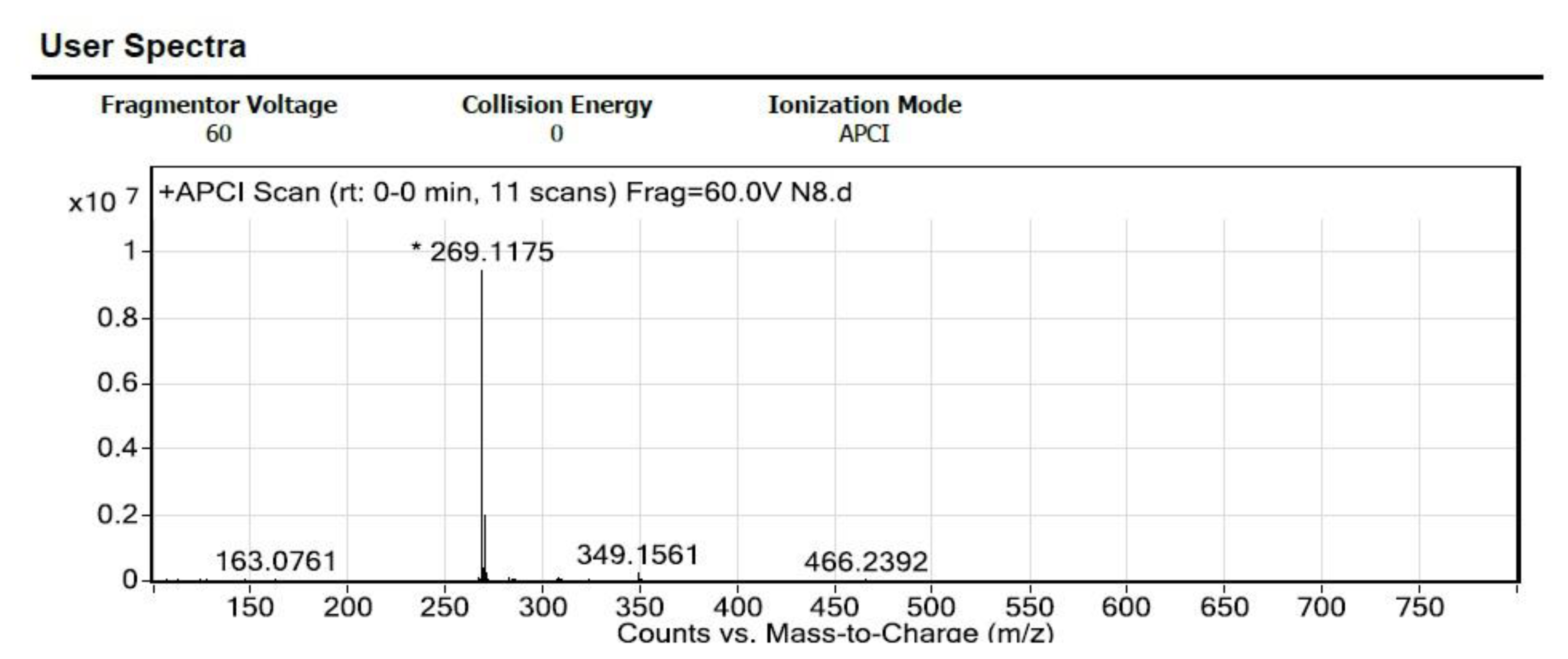

Supplement: Figure 24 — HRMS spectrum of (E)-1,5-bis(4-hydroxyphenyl)pent-1-en-3-one (5h). (C17H16O3+H)+, Calc: 269.1177. [file turkjchem-47-5-1249s24.tif]

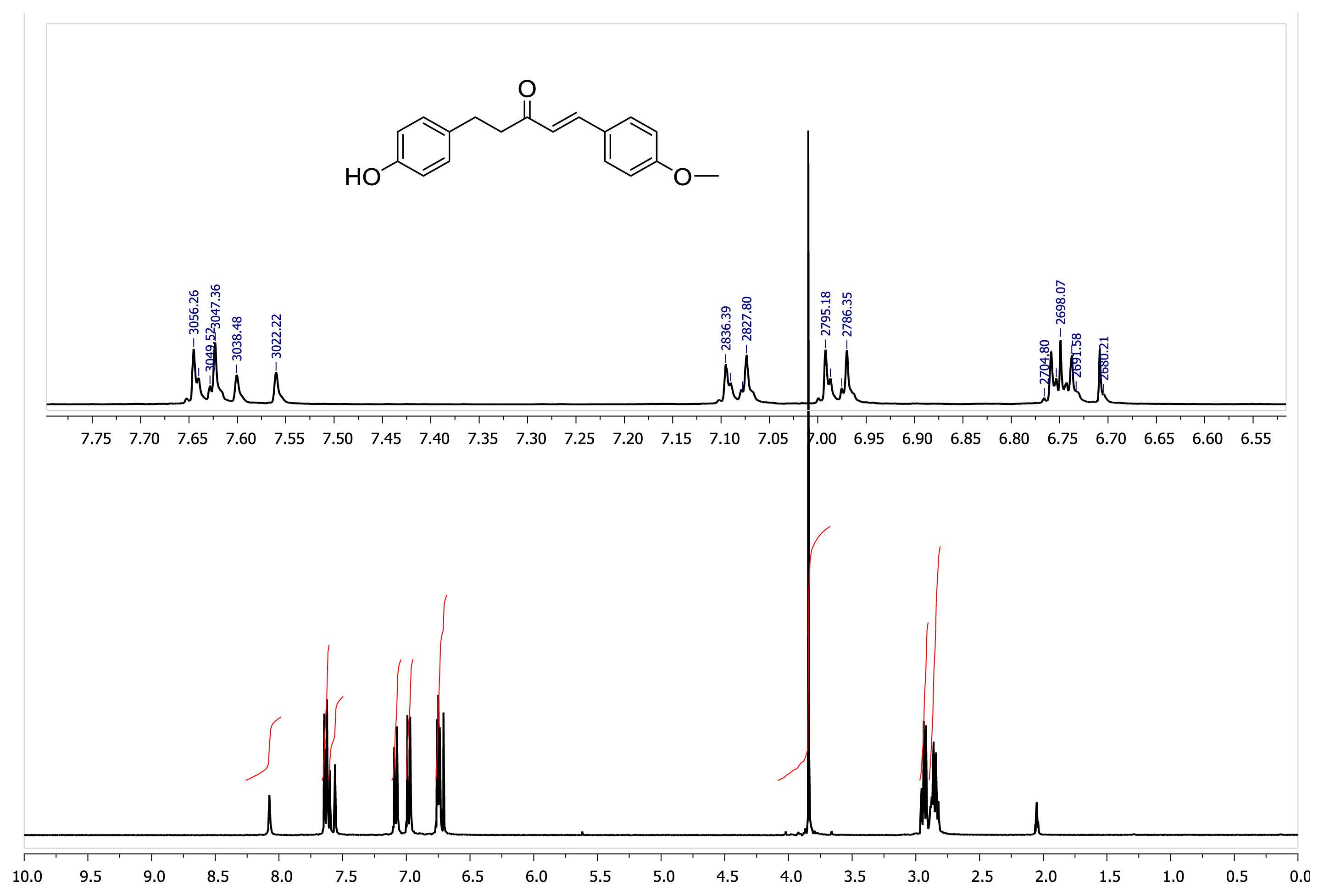

Supplement: Figure 25 — 1H-NMR spectrum of (E)-5-(4-hydroxyphenyl)-1-(4-methoxyphenyl)pent-1-en-3-one (5i) (Acetone-d6). [file turkjchem-47-5-1249s25.tif]

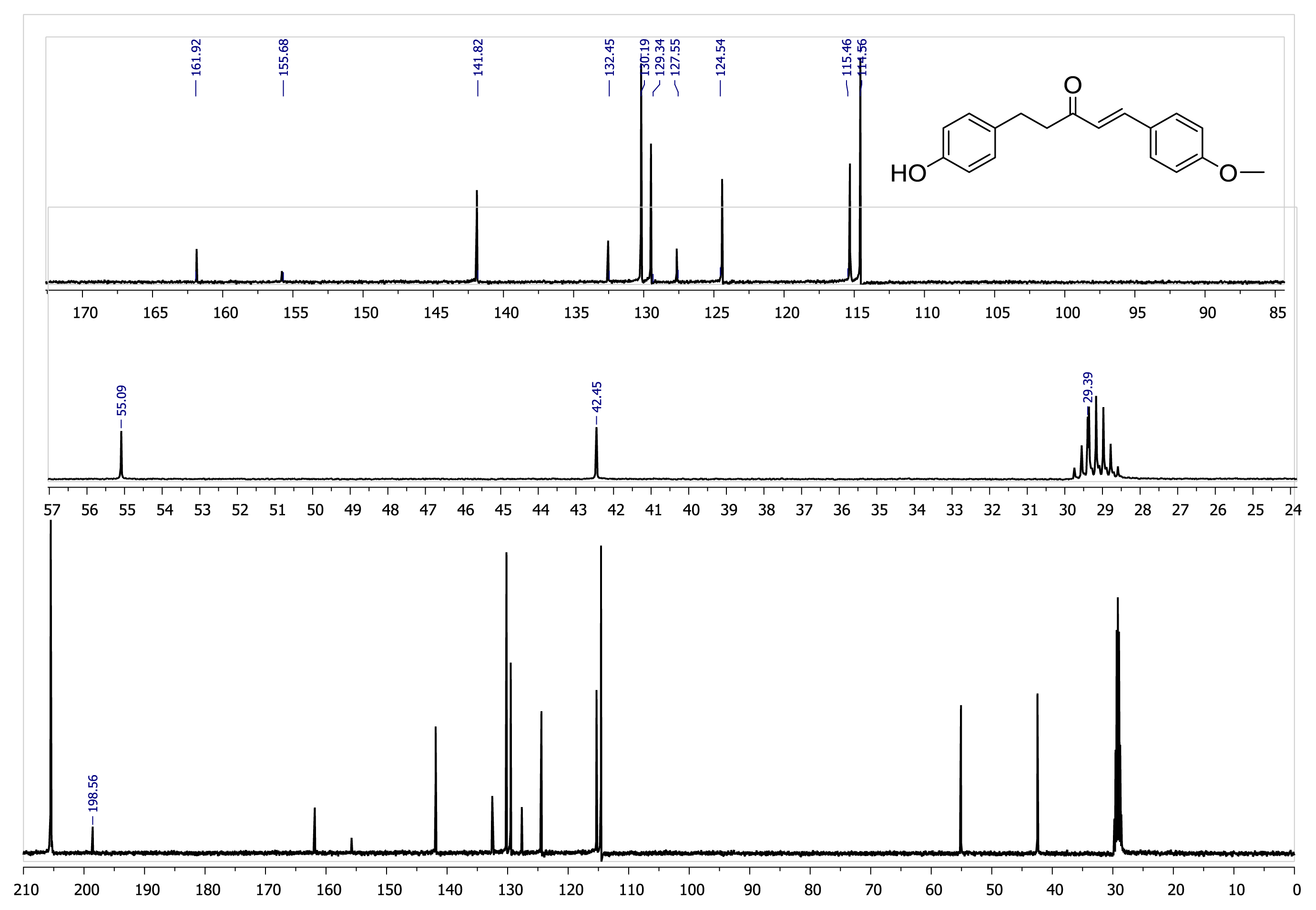

Supplement: Figure 26 — 13C-NMR spectrum of (E)-5-(4-hydroxyphenyl)-1-(4-methoxyphenyl)pent-1-en-3-one (5i) (Acetoned6). [file turkjchem-47-5-1249s26.tif]

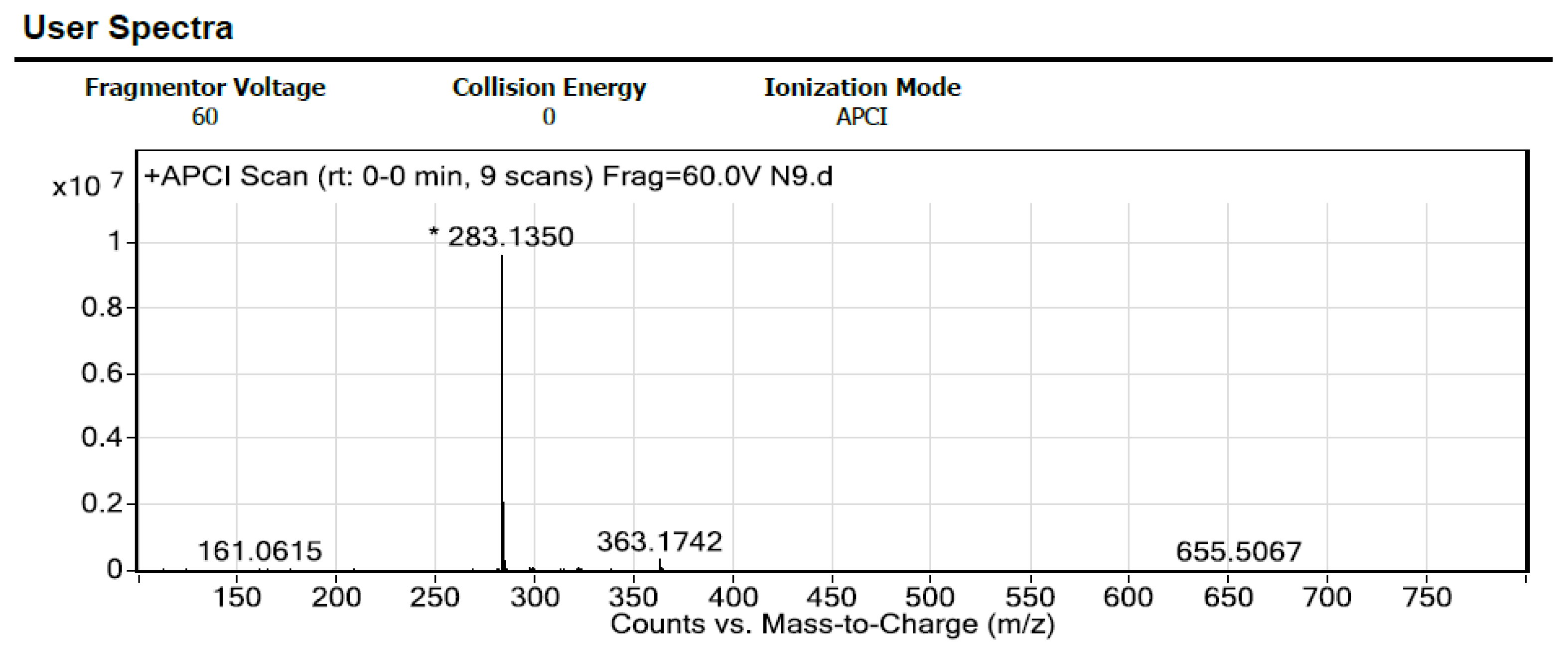

Supplement: Figure 27 — HRMS spectrum of (E)-5-(4-hydroxyphenyl)-1-(4-methoxyphenyl)pent-1-en-3-one (5i). (C18H18O3+H)+, Calc: 283.1334. [file turkjchem-47-5-1249s27.tif]

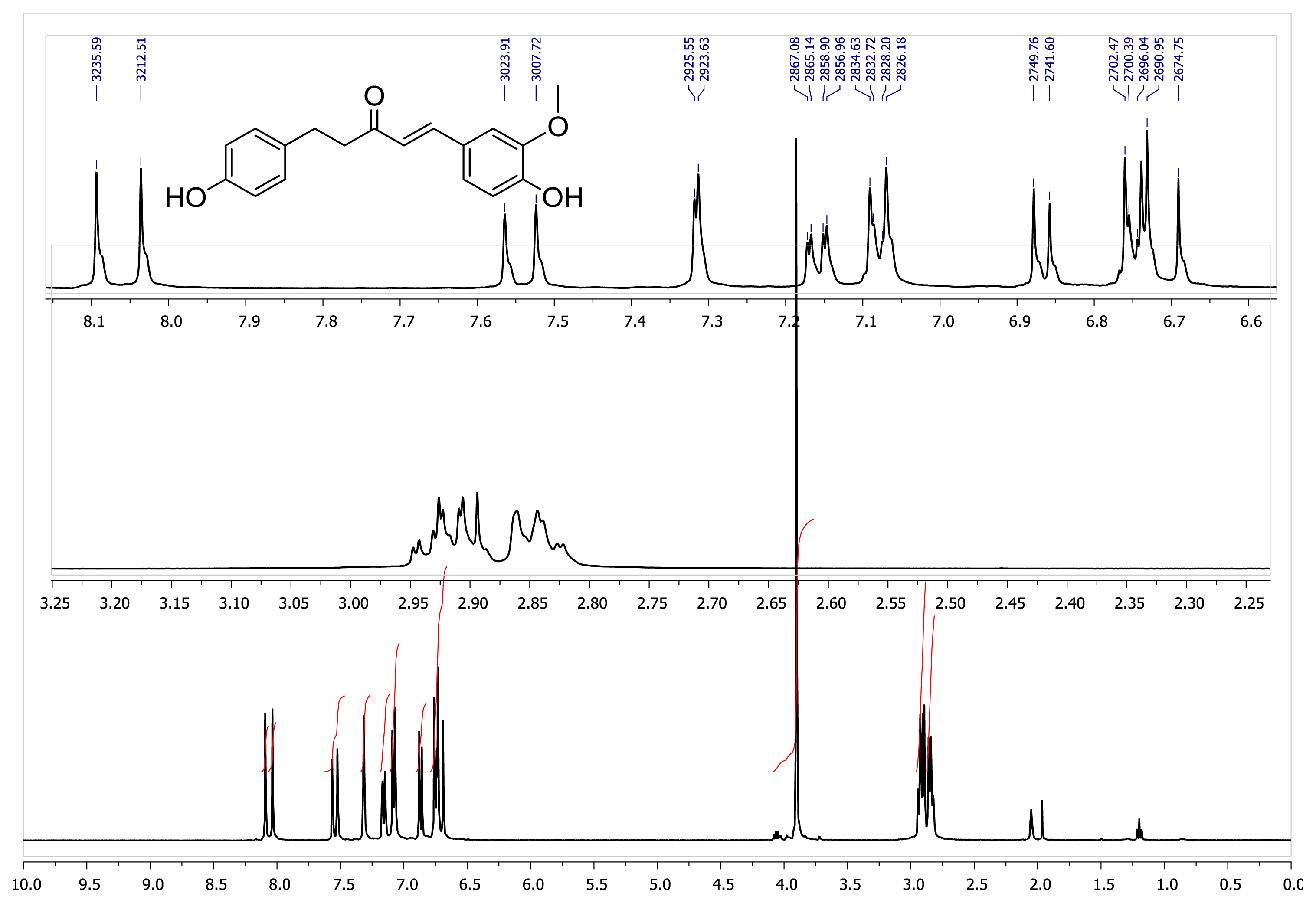

Supplement: Figure 28 — 1H-NMR spectrum of (E)-1-(4-hydroxy-3-methoxyphenyl)-5-(4-hydroxyphenyl)pent-1-en-3-one (5j) (Acetone-d6). [file turkjchem-47-5-1249s28.tif]

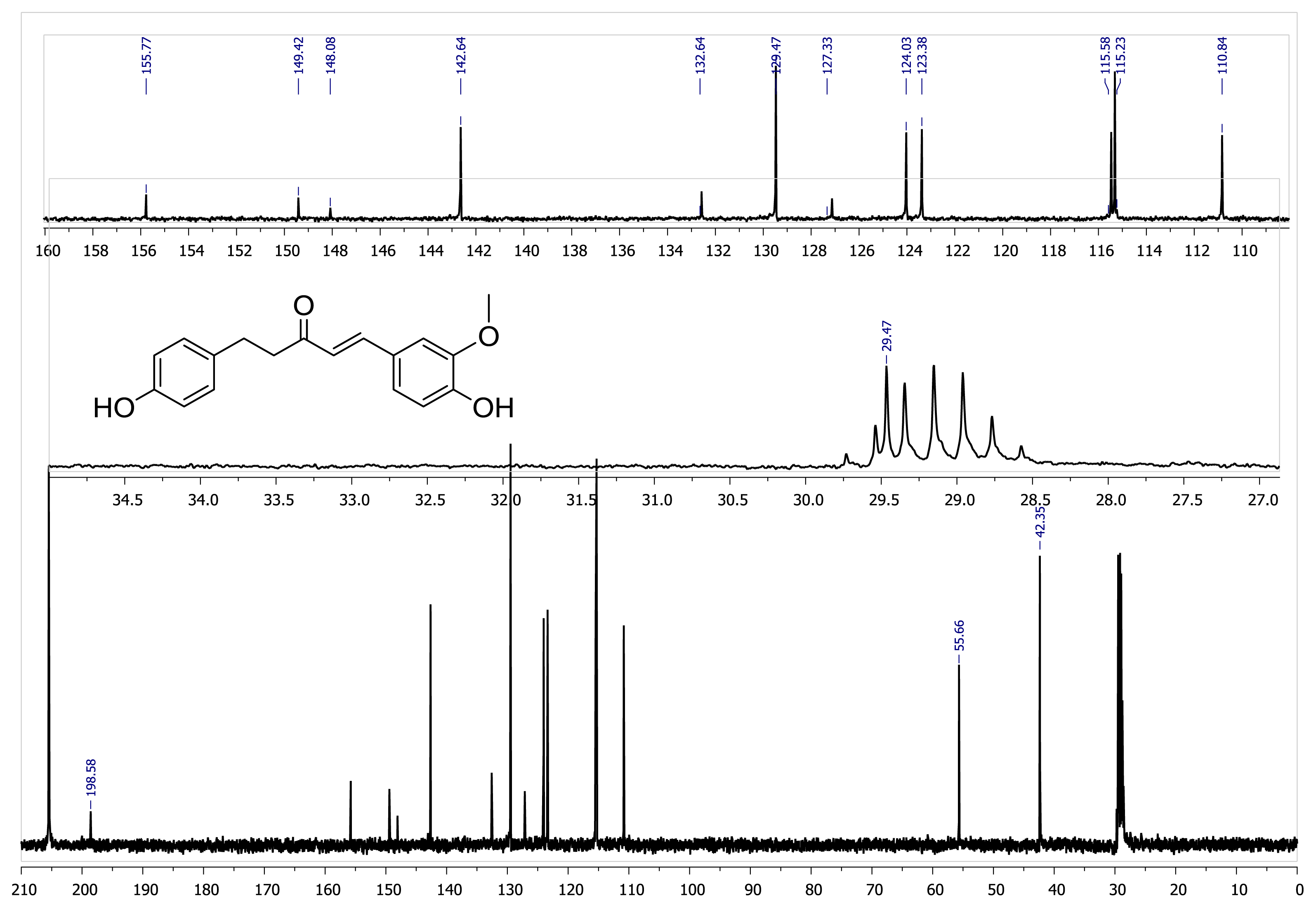

Supplement: Figure 29 — 13C-NMR spectrum of (E)-1-(4-hydroxy-3-methoxyphenyl)-5-(4-hydroxyphenyl)pent-1-en-3-one (5j) (Acetone-d6). [file turkjchem-47-5-1249s29.tif]

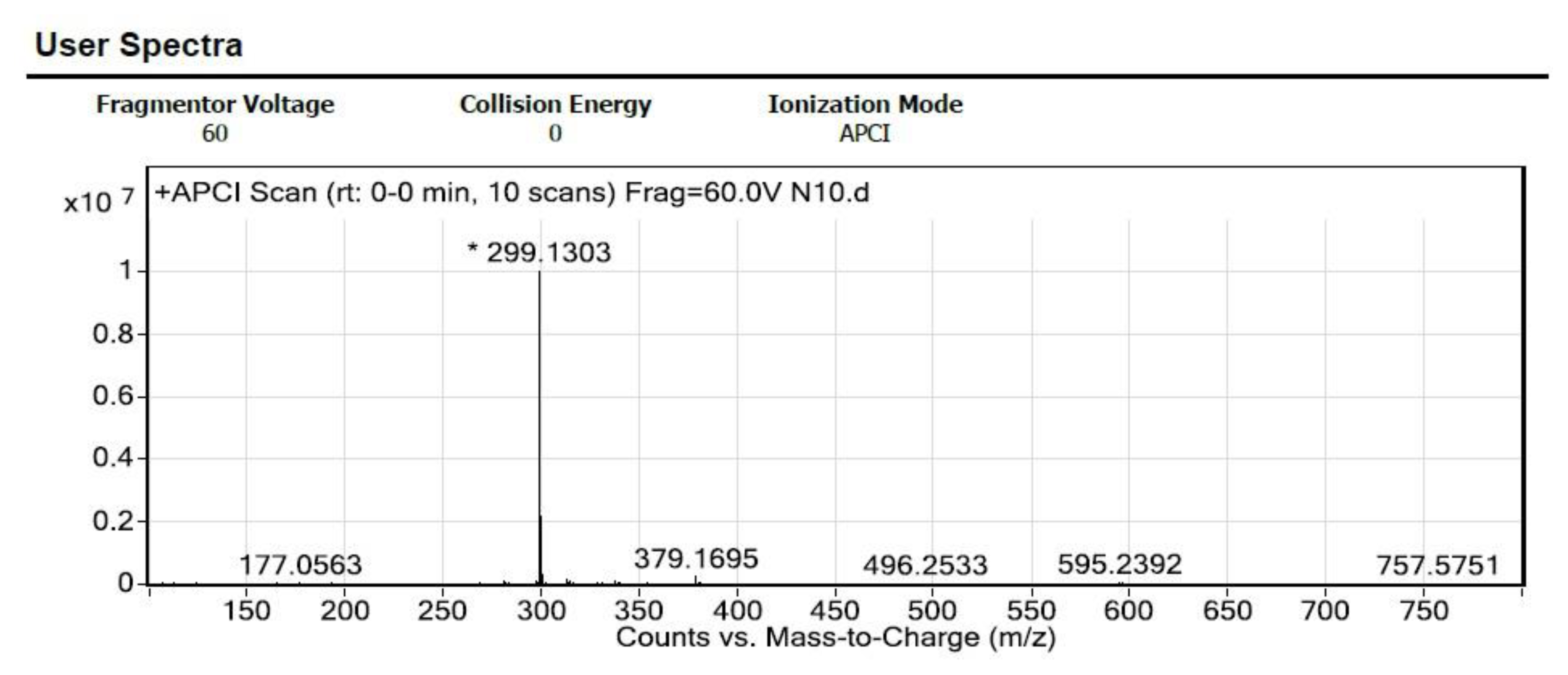

Supplement: Figure 30 — HRMS spectrum of (E)-1-(4-hydroxy-3-methoxyphenyl)-5-(4-hydroxyphenyl)pent-1-en-3-one (5j). (C18H18O4+H)+, Calc: 299.1283. [file turkjchem-47-5-1249s30.tif]

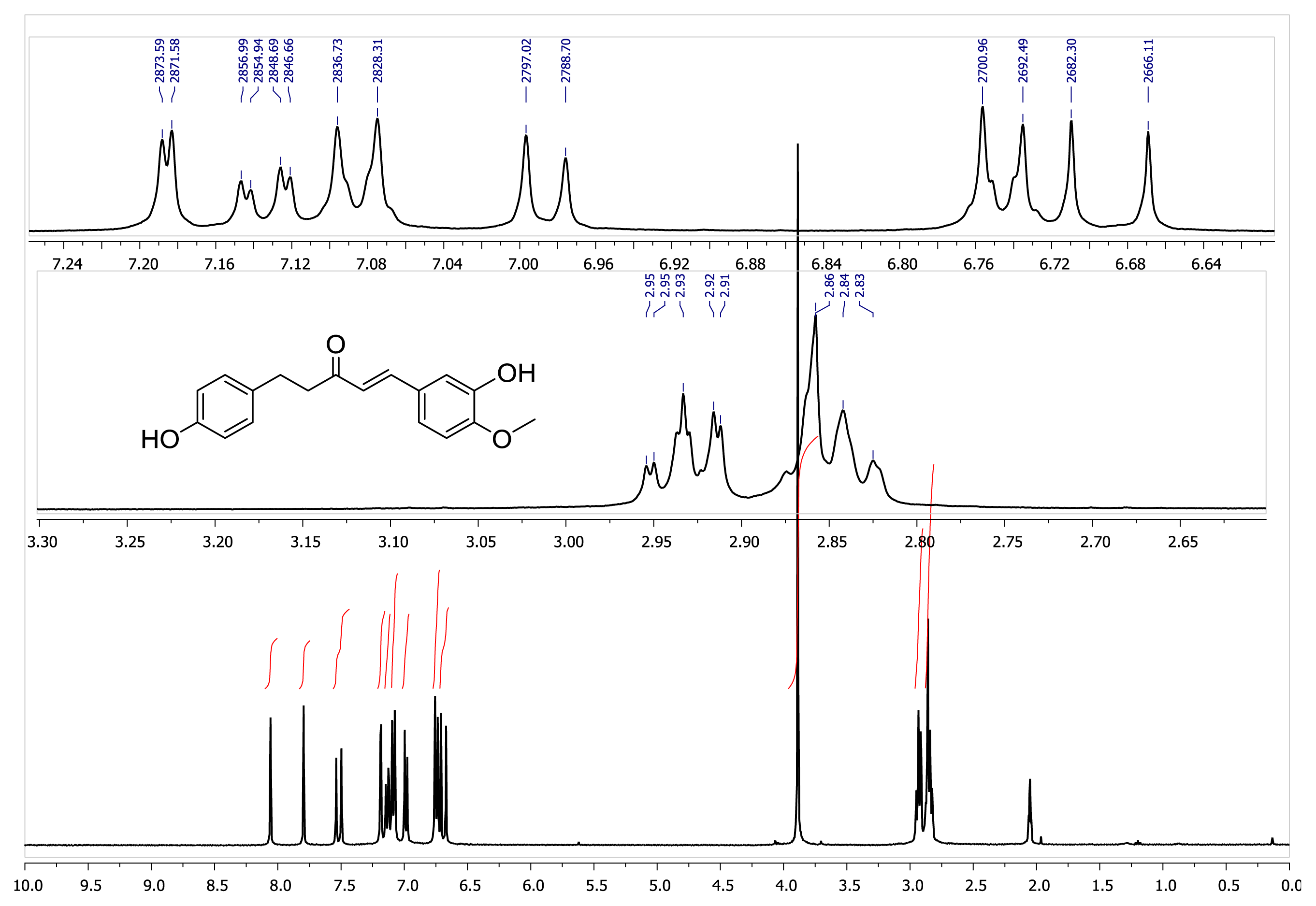

Supplement: Figure 31 — 1H-NMR spectrum of (E)-1-(3-hydroxy-4-methoxyphenyl)-5-(4-hydroxyphenyl)pent-1-en-3-one (5k) (Acetone-d6). [file turkjchem-47-5-1249s31.tif]

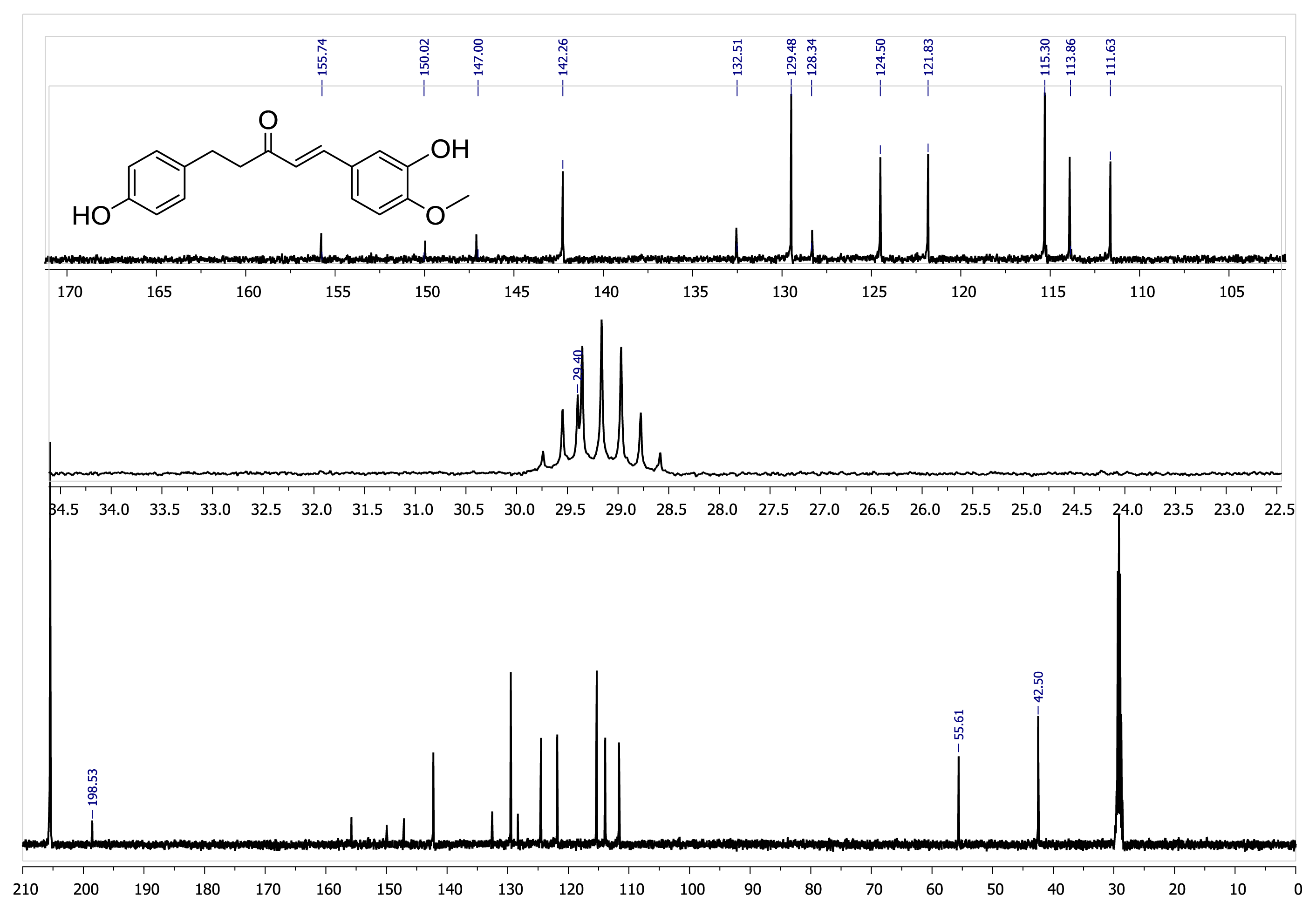

Supplement: Figure 32 — 13C-NMR spectrum of (E)-1-(3-hydroxy-4-methoxyphenyl)-5-(4-hydroxyphenyl)pent-1-en-3-one (5k) (Acetone-d6). [file turkjchem-47-5-1249s32.tif]

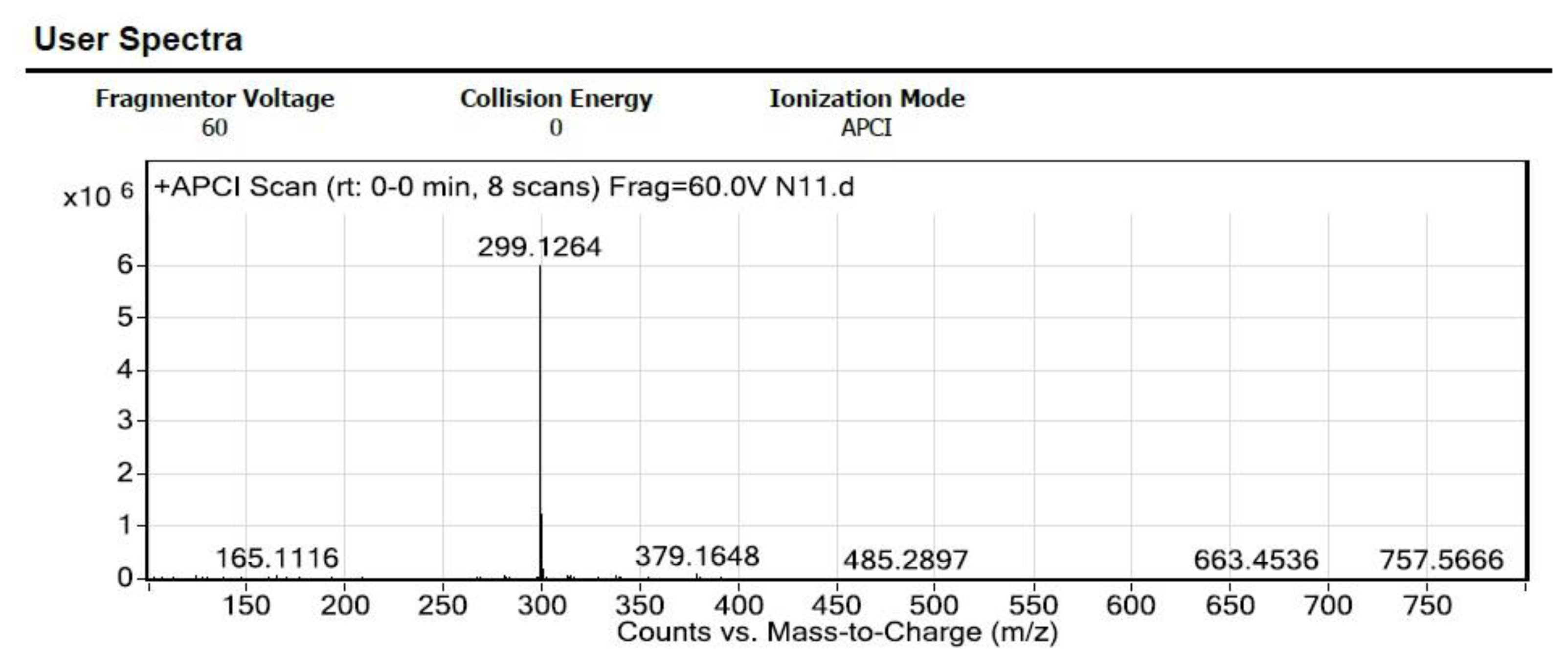

Supplement: Figure 33 — HRMS spectrum of (E)-1-(3-hydroxy-4-methoxyphenyl)-5-(4-hydroxyphenyl)pent-1-en-3-one (5k). (C18H18O4+H)+, Calc: 299.1283. [file turkjchem-47-5-1249s33.tif]

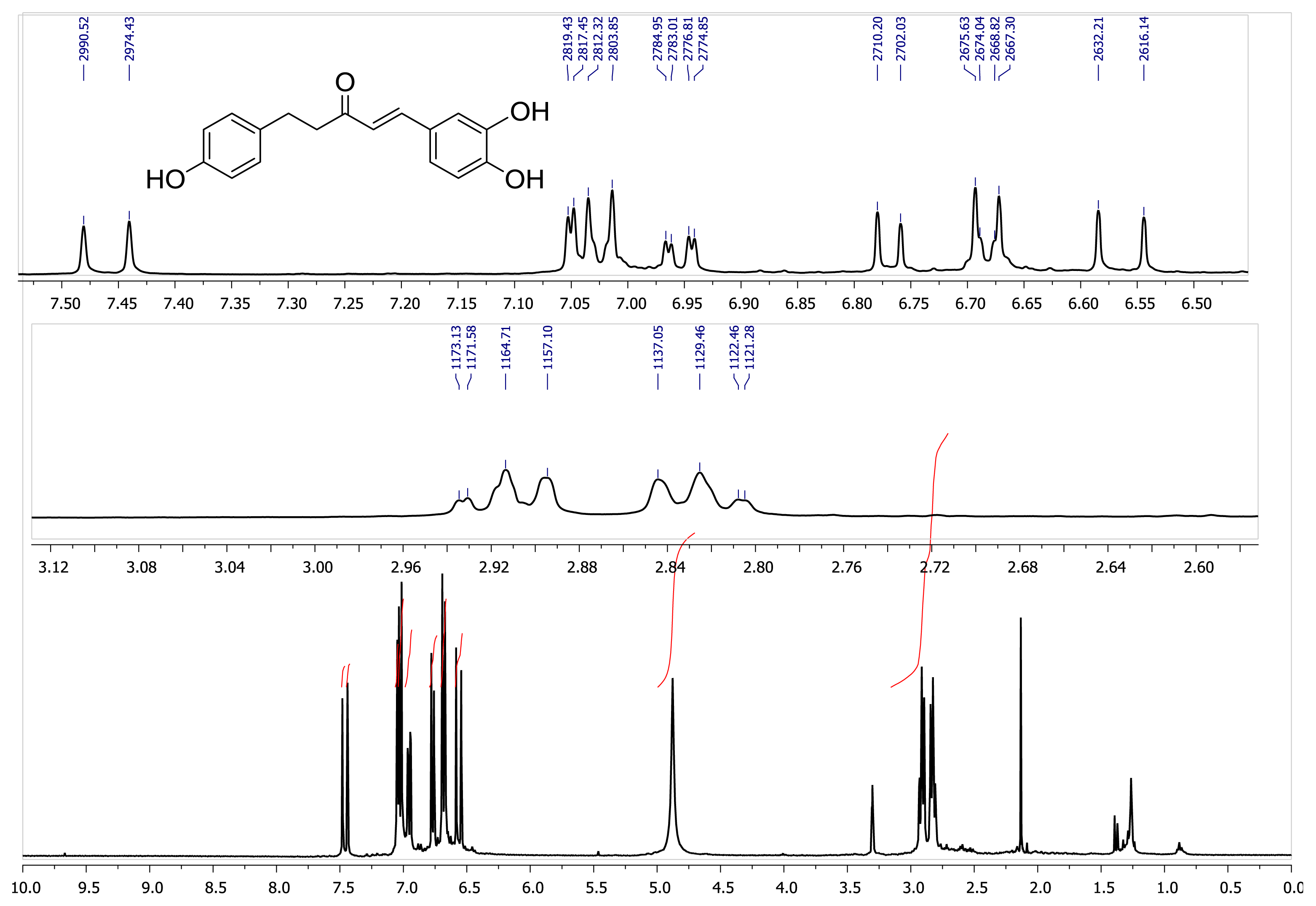

Supplement: Figure 34 — 1H-NMR spectrum of (E)-1-(3,4-dihydroxyphenyl)-5-(4-hydroxyphenyl)pent-1-en-3-one (5l) (Methanol-d4). [file turkjchem-47-5-1249s34.tif]

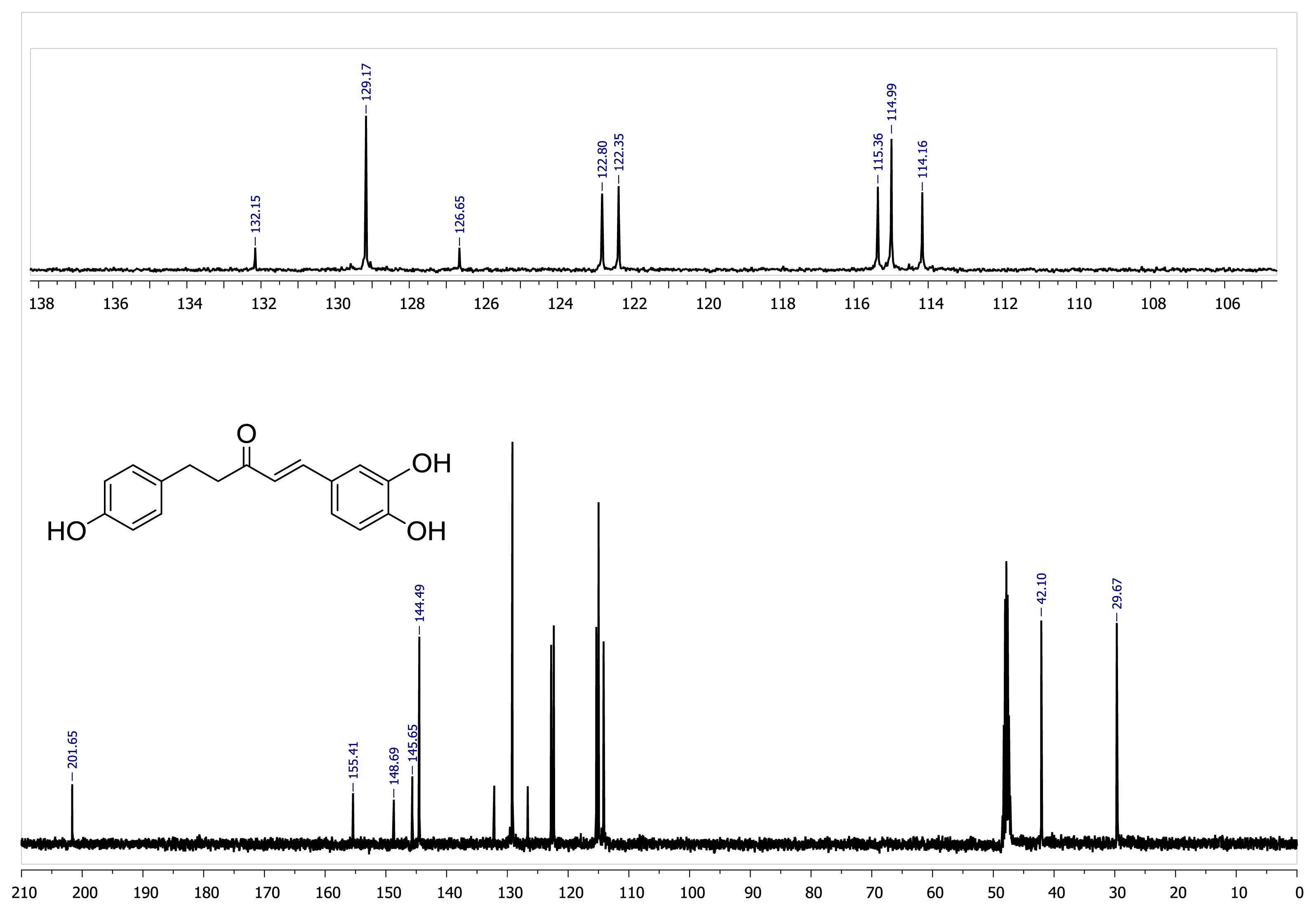

Supplement: Figure 35 — 13C-NMR spectrum of (E)-1-(3,4-dihydroxyphenyl)-5-(4-hydroxyphenyl)pent-1-en-3-one (5l) (Methanol-d4). [file turkjchem-47-5-1249s35.tif]

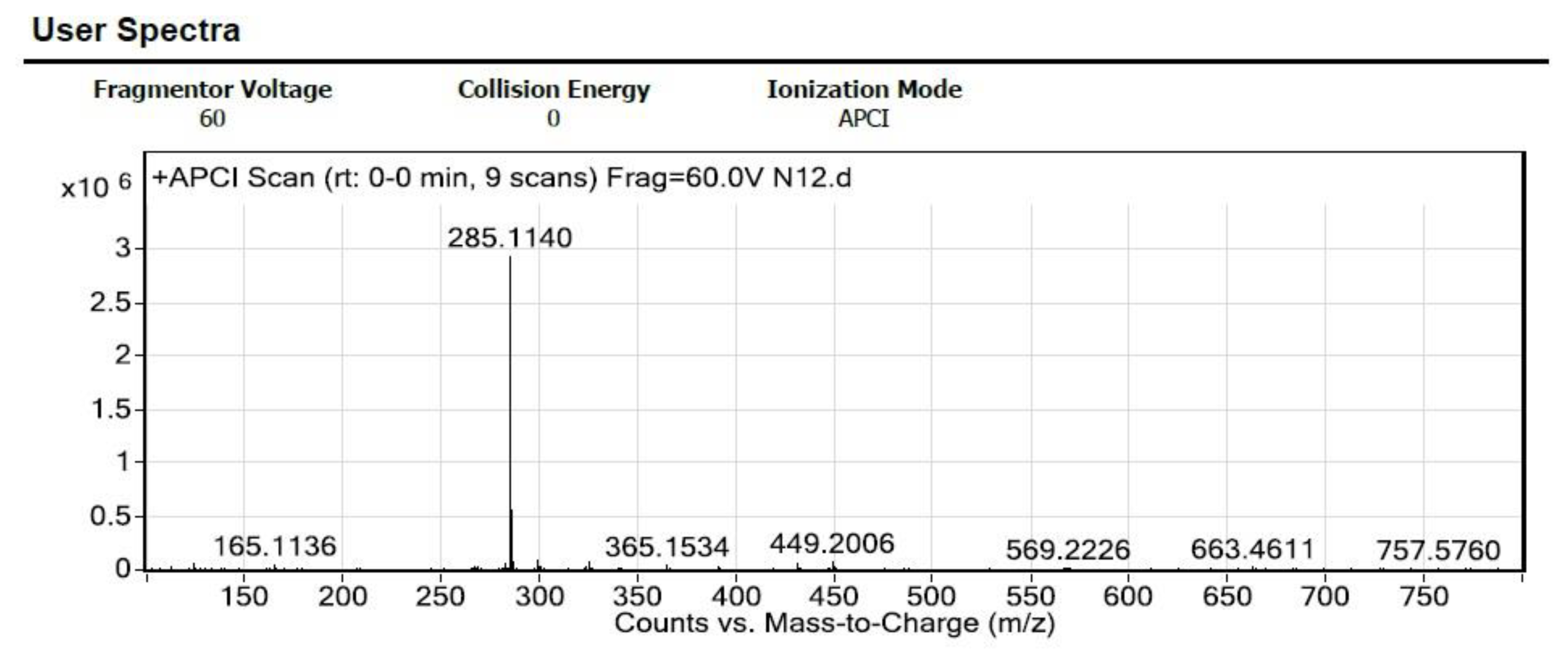

Supplement: Figure 36 — HRMS spectrum of (E)-1-(3,4-dihydroxyphenyl)-5-(4-hydroxyphenyl)pent-1-en-3-one (5l). (C17H16O4+H)+, Calc: 285.1126. [file turkjchem-47-5-1249s36.tif]
